# The deletion of AQP4 and TRPV4 affects astrocyte swelling/volume recovery in response to ischemia-mimicking pathologies

**DOI:** 10.3389/fncel.2024.1393751

**Published:** 2024-05-15

**Authors:** Zuzana Hermanova, Lukas Valihrach, Jan Kriska, Mansi Maheta, Jana Tureckova, Mikael Kubista, Miroslava Anderova

**Affiliations:** ^1^Department of Cellular Neurophysiology, Institute of Experimental Medicine CAS, Prague, Czechia; ^2^Second Faculty of Medicine, Charles University, Prague, Czechia; ^3^Laboratory of Gene Expression, Institute of Biotechnology CAS, Vestec, Czechia

**Keywords:** aquaporin 4, transient receptor potential vanilloid 4, astrocytes, brain edema, ischemia

## Abstract

**Introduction:**

Astrocytic Transient receptor potential vanilloid 4 (TRPV4) channels, together with Aquaporin 4 (AQP4), are suspected to be the key players in cellular volume regulation, and therefore may affect the development and severity of cerebral edema during ischemia. In this study, we examined astrocytic swelling/volume recovery in mice with TRPV4 and/or AQP4 deletion in response to *in vitro* ischemic conditions, to determine how the deletion of these channels can affect the development of cerebral edema.

**Methods:**

We used three models of ischemia-related pathological conditions: hypoosmotic stress, hyperkalemia, and oxygenglucose deprivation (OGD), and observed their effect on astrocyte volume changes in acute brain slices of Aqp4^–/–^, Trpv4^–/–^ and double knockouts. In addition, we employed single-cell RT-qPCR to assess the effect of TRPV4 and AQP4 deletion on the expression of other ion channels and transporters involved in the homeostatic functioning of astrocytes.

**Results:**

Quantification of astrocyte volume changes during OGD revealed that the deletion of AQP4 reduces astrocyte swelling, while simultaneous deletion of both AQP4 and TRPV4 leads to a disruption of astrocyte volume recovery during the subsequent washout. Of note, astrocyte exposure to hypoosmotic stress or hyperkalemia revealed no differences in astrocyte swelling in the absence of AQP4, TRPV4, or both channels. Moreover, under ischemia-mimicking conditions, we identified two distinct subpopulations of astrocytes with low and high volumetric responses (LRA and HRA), and their analyses revealed that mainly HRA are affected by the deletion of AQP4, TRPV4, or both channels. Furthermore, gene expression analysis revealed reduced expression of the ion transporters KCC1 and ClC2 as well as the receptors GABA_B_ and NMDA in Trpv4^–/–^ mice. The deletion of AQP4 instead caused reduced expression of the serine/cysteine peptidase inhibitor Serpina3n.

**Discussion:**

Thus, we showed that in AQP4 or TRPV4 knockouts, not only the specific function of these channels is affected, but also the expression of other proteins, which may modulate the ischemic cascade and thus influence the final impact of ischemia.

## 1 Introduction

Astrocytes comprise the most numerous types of glial cells, which in the healthy brain possess a wide range of functions, e.g., the maintenance of ionic and neurotransmitter homeostasis, water transport, pH regulation, energy supply for neurons, and local blood flow regulation (Sofroniew and Vinters, 2010; Vasile et al., 2017). Moreover, the astrocytic processes surrounding the blood vessels act as part of the blood-brain barrier (BBB), and form glia limitans around the whole brain. As they possess multiple roles in the healthy tissue, inevitably, they are also key players in numerous pathological processes – brain injuries, tumors, and neurodegenerative diseases (Vasile et al., 2017; Filipi et al., 2020; Habib et al., 2020). Cerebral edema represents a serious complication in various brain disorders, such as ischemic or traumatic injury or tumor growth. Astrocytes represent the key contributors to the development of a vasogenic type of edema, occurring after BBB disruption and characterized by water accumulation in extracellular space (ECS), as well as a cytotoxic (cellular) type of edema characterized by cellular swelling [for a review see (Wilson and Mongin, 2018; Jha et al., 2019)]. During brain edema development, astrocytes take up ions and water, and thus they increase their volume. Swollen astrocytes then exert pressure on the surrounding tissue and compromise blood flow, creating additional local ischemic lesions (Simard et al., 2007). Astrocytic swelling is a complex process, which includes the activation of a large number of membrane channels and transporters, including Na^+^/K^+^/Cl^–^ cotransporter 1 (NKCC1), inwardly-rectifying potassium channels (Kir) and voltage-gated chloride channels (ClC) or K^+^/Cl^–^ cotransporters (KCC), which mediate the transport of osmotically active solutes (K^+^, Na^+^, Cl^–^ or Ca^2+^) across the plasmatic membranes of astrocytes (Ernest et al., 2005; Pasantes-Morales and Vázquez-Juárez, 2012; Dijkstra et al., 2016; Larsen and MacAulay, 2017; Stokum et al., 2018; Formaggio et al., 2019; Lafrenaye and Simard, 2019; Wilson et al., 2019). Water follows these solutes through aquaporin 4 (AQP4) channels and, as a result, the cells swell (Stokum et al., 2018; Lafrenaye and Simard, 2019). The solutes and water then move through the astrocytic syncytium via connexin hemichannels. The increase in astrocytic volume during swelling, and the subsequent membrane stretch, activates the transient receptor potential vanilloid 4 (TRPV4) channels, volume-regulated anion channels (VRACs), several members of the ClC family, Ca^2+^-activated chloride channels (CaCC), Kir and others, and their activation consequently leads to the efflux of ions from the astrocytic cytoplasm (Jo et al., 2015; Okada et al., 2019). Water then follows the ions through the AQP4 channels to reach the osmotic equilibrium, and the whole process results in an astrocytic regulatory volume decrease (RVD) (Benfenati et al., 2011; Mola et al., 2016).

AQP4 channels represent the main water pathway within the astrocytes and manage water flow through the BBB. They are predominantly expressed on perivascular astrocytic endfeet. This specific polarized localization is dependent on the expression of different AQP4 isoforms (Lisjak et al., 2020) that interact with the α-syntrophin and dystrophin complex. The deletion of the α-syntrophin complex alters the astrocytic swelling during numerous pathological stimulations (Dmytrenko et al., 2013; Anderova et al., 2014). The density of AQP4 channels on astrocytic membranes varies in the pathological environment due to their redistribution within the cells, as well as the stimulation/attenuation of AQP4 expression, which strongly affects the dynamics of water transport in the brain parenchyma (Lisjak et al., 2020). Moreover, AQP4 expression and its localization are affected by several channels and proteins, such as TRPV4 channels (Salman et al., 2017), NKCC1 (Jayakumar and Norenberg, 2010) and calmodulin (Kitchen et al., 2016, 2020). It was suggested that astrocytes function as intracranial baroreceptors, and in this process, TRPV4 channels are essential as part of the astrocytic mechanosensory signaling (Shibasaki, 2016; Turovsky et al., 2020). This hypothesis was further supported by the data from *in vitro* experiments, which showed that TRPV4 and AQP4 channels colocalize. Their functional cooperation was described in astrocytic cultures and in cultured retinal Müller glia (Benfenati et al., 2011; Jo et al., 2015). Moreover, the blockage of both AQP4 and TRPV4 attenuates the development of brain edema and BBB disruption (Jie et al., 2015). In addition, the involvement of AQP4 and TRPV4 channels in RVD has been proposed in several studies (Benfenati et al., 2011; Jo et al., 2015; Iuso and Križaj, 2016). An understanding of the role of both channels in astrocyte volume regulation is therefore essential for the development of new, more effective treatments for brain edema.

In this study, we aim to contribute to the understanding of the astrocytic role in ischemic brain injury and the development of brain edema. We used genetically modified mice with deleted AQP4, TRPV4, or both channels. The global knockouts were crossbred with GFAP/EGFP mice with visualized astrocytes to elucidate the role of both channels in astrocytic swelling, and their cooperation in the regulation of astrocytic volume. The acute brain slices were subjected to different ischemia-related pathological stimuli, such as hypoosmotic stress, oxygen-glucose deprivation (OGD), or hyperkalemia, and a fluorescence-based approach was used to quantify the volume of single astrocytes. To gain a greater understanding of the processes underlying astrocytic swelling, we used single-cell gene expression profiling of control (Ctrl) and knockout animals to disclose changes in the expression of genes that can play a role in the swelling and the regulation of astrocyte volume and therefore in brain edema development.

## 2 Materials and methods

### 2.1 Experimental animals

Our experiments were performed on adult (90 ± 10 days old) transgenic mice with fluorescently labeled astrocytes (line designation TgN(GFAP-EGFP), FVB background), in which the expression of enhanced green fluorescent protein (EGFP) was controlled by the human glial fibrillary acidic protein (*Gfap*) promoter (Nolte et al., 2001). These animals were either cross-bred with the *Trpv4*-deficient strain (Trpv4^–/–^; on C57BL/6N background) with excised exon 12 encoding transmembrane pore domains 5 and 6 (Liedtke and Friedman, 2003) or with *Aqp4*-deficient (Aqp4^–/–^) mice. They were generated as previously described by Ikeshima-Kataoka et al. (2013), and frozen embryos were obtained from Riken BRC (acc. no. CDB0758K-1;^1^ genetic background B6 mixed with Balb/c), where breeding lines were established through embryo transfer. Homozygous *Trpv4*- and *Aqp4*-deficient lines were established together with animal lines deficient in both TRPV4 and AQP4 (Aqp4^–/–^/Trpv4^–/–^) and the homozygous *Aqp4*- and *Trpv4*-positive line was used as Ctrl.

All procedures involving the use of laboratory animals were performed in accordance with the European Communities Council Directive 24 November 1986 (86/609/EEC) and animal care guidelines approved by the Institute of Experimental Medicine, Czech Academy of Sciences (Animal Care Committee on April 30, 2019; approval number 49/2019). All efforts were made to minimize both the suffering and the number of animals used.

### 2.2 Experimental solutions

The composition of the artificial cerebrospinal fluid (aCSF), isolation solution, 200 mOsmol hypotonic solution (H-100), hyperkalemic solution (50 mM K^+^) and solution for OGD is listed in Table 1. All solutions except the OGD were equilibrated with 95 % O_2_ and 5 % CO_2_ (Carbogen; Siad, Branany, Czech Republic) to the final pH of 7.4 and osmolality was measured using a vapor pressure osmometer (Vapro 5520, Wescor, Logan, UT). The OGD solution was saturated with 5 % O_2_, 90 % N_2_ and 5 % CO_2_.

**TABLE 1:** Contents of experimental solutions.

Compounds	aCSF [mM]	Isolation solution [mM]	H-100 [mM]	50 mM K^+^ [mM]	OGD [mM]	Isolation buffer [mM]
NaCl	122	–	67	75	122	136
NMDG	–	110	–	–	–	–
KCl	3	2.5	3	50	3	5.4
NaHCO_3_	28	24.5	28	28	28	–
Na_2_HPO_4_	1.25	1.25	1.25	1.25	1.25	–
Glucose	10	20	10	10	–	5.5
CaCl_2_	1.5	0.5	1.5	1.5	1.5	–
MgCl_2_	1.3	7	1.3	1.3	1.3	–
HEPES	–	–	–	–	–	10
Osmolality (mOsmol/kg)	∼300	∼300	∼200	∼300	∼300	∼290

NMDG, N-methyl-D-glucamine; aCSF, artificial cerebrospinal fluid; H-100, 200 mOsmol hypotonic solution; OGD, oxygen-glucose deprivation; 50 mM K^+^, artificial cerebrospinal fluid with elevated K^+^ concentration.

### 2.3 Acute brain slice preparation

Animals were anesthetized using an intraperitoneal injection of pentobarbital (PTB; 100 mg/kg), and transcardially perfused with a cold (4 ± 1°C) isolation solution. Perfused animals were decapitated, and the brains were removed. Acute coronal slices (300 μm) were prepared from each brain on a vibrating microtome (Leica VT 1200S; Baria s.r.o., Czech Republic). The slices were incubated in preheated (34°C) isolation solution for 30 min and then for another 30 min at room temperature in aCSF. Before microscopic scanning, every slice was incubated for 10 min in preheated aCSF (34°C) and kept at 32 ± 1°C during the measurements.

### 2.4 Image acquisition using two-photon microscopy

**FIGURE 1:**
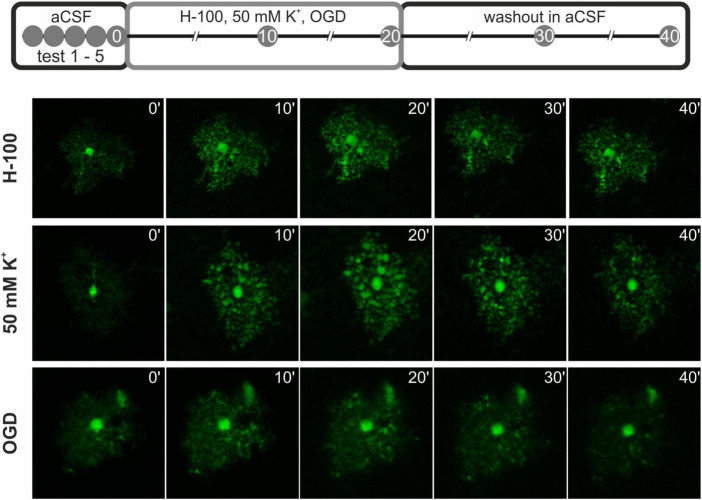
Scheme of the pathological stimuli application during astrocytic volume measurements. At first, 5 test measurements were made (test 1 – 5) to assess photobleaching. Next, the pathological stimulus - 200 mOsmol hypotonic solution (H-100), oxygen-glucose deprivation (OGD), or hyperkalemic solution (50 mM K^+^) was applied for 20 min, and the astrocytic volume was measured every 10 min. Finally, a washout phase in artificial cerebrospinal fluid (aCSF) was performed for another 20 min with measurements every 10 min. Below, representative images of the cells at each time point after the application of the pathological stimuli and during washout are presented.

Fluorescence images were acquired from cortex in acute brain slices using a multiphoton laser scanning microscope FV1200MPE (Olympus) with 60x LUMPLFLN water objective. The fluorescence of EGFP was excited by 950 nm and the signal was detected using appropriate emission filters. Whole cortical astrocytes were recorded as a set of images (Z-stack) with a constant spacing/step size of 0.5 μm. Approximately 150 – 200 focal images were acquired for every cell, based on the size of each cell. The imaged area was extended to accommodate not only the desired cell, but also space around to avoid losing the cell due to the swelling of the tissue and subsequent movement in x/y axes, as well as z-position of the cells. The area for calculating the change in astrocytic soma volume was then defined manually. Five Z-stacks were acquired before the experiment for the correction of scanning photobleaching (initial testing). Subsequently, two sets of images were acquired 10 and 20 min after the application of pathological stimulation (Table 1), and another two sets of images were acquired after the repeated application of aCSF (washout; Figure 1; Awadová et al., 2018; Pivonkova et al., 2018).

### 2.5 The quantification of cellular volume changes in acute brain slices

**FIGURE 2:**
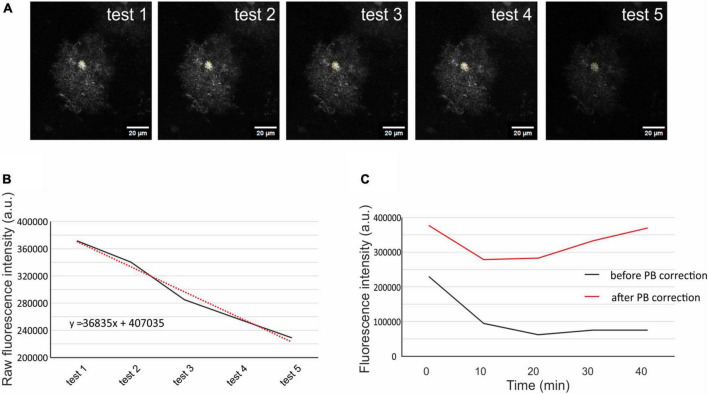
Correction of scanning photobleaching. From the 5 initial tests average fluorescence intensity was measured in a circular selection in the cell soma **(A)**. The raw intensity of the fluorescence was put into a graph, and linear trendline was added (red dotted line, **B**). The trendline equation was used to estimate the fluorescence decay of each measured cell. The original fluorescence intensity (**C**; black line) was corrected using the trendline equation and the corrected fluorescence intensity (**C**; red line) was then used for the quantification of the cell volume. The representative cell chosen for this figure is from Aqp4^–/–^ mouse strain. Aqp4^–/–^, Aquaporin 4-deficient; PB, photobleaching.

The quantification of cellular volume changes was performed using a fluorescence-intensity approach in the cell soma as previously described (Awadová et al., 2018; Pivonkova et al., 2018). Image processing and morphometry measurements were performed using the ImageJ/Fiji software.^2^ Average fluorescence intensity projections of cell somas from the Z-stacks were created for each cell during the initial testing (5 time points), pathological stimulation (2 time points), and the subsequent washout phase (2 time points, Figure 1). These projections were used for creating another stack of images, where the somas from each projection were superimposed on top of each other. The average intensity of fluorescence was measured from circular selection from the cell soma (the selection was the same through all the time points of one cell). The raw intensity of the fluorescence was corrected for scan-induced photobleaching, using linear estimation of fluorescence decay. Since the fluorescence intensity decreased proportionally to the swelling of the cells, we counted the cellular volume as 1/fluorescence intensity (Figure 2; for more information see our previously published methodical article by Awadová et al. (2018). The astrocyte volume at *t* = 0 min (5^th^ measurement of initial testing) was set to 100 % and the astrocytic volume changes were expressed relative to this baseline as an increase/decrease in percentage. In total, 217 cells from 48 mice were analyzed: 14 Ctrl mice (48 cells), 11 Aqp4^–/–^ mice (56 cells), 14 Trpv4^–/–^ mice (60 cells), and 9 Aqp4^–/–^/Trpv4^–/–^ mice (53 cells). The data are presented as the mean ± SEM. Statistical analyses of the differences in astrocytic volume among groups were performed using ANOVA for multiple comparisons with Bonferroni’s *post hoc* test. Differences between the groups were considered statistically significant when *p* < 0.05, very significant when *p* < 0.01, and extremely significant when *p* < 0.001.

### 2.6 The preparation and collection of single cells for gene expression profiling

The mice were anesthetized with PTB (100 mg/kg, i.p.) and perfused transcardially with a cold (4 ± 1°C) isolation buffer (Table 1). The cortex was removed and used for the preparation of cell suspension using a papain dissociation kit (Worthington, NJ, USA). The cell suspension was layered on top of 5 ml of Ovomucoid inhibitor solution (Worthington, Lakewood, NJ, USA) and the cells were subsequently harvested by centrifugation (140× *g* for 6 min). Cell aggregates were removed by filtering with 70 μm cell strainers (Becton Dickinson, NJ, USA), and the cells were kept on ice until sorting. The EGFP-positive astrocytes were collected using fluorescence-activated cell sorting (FACS; BD Influx, San Jose, CA, USA). FACS was calibrated manually to deposit single cells in the center of each collection tube. Hoechst 33258 (Life Technologies, Carlsbad, CA, USA) was added to the suspension of cells to check the cell viability. Single cells were sorted into 96-well plates (Life Technologies, Carlsbad, CA, USA), each well contained 5 μl of nuclease-free water with bovine serum albumin (1 mg/ml, Fermentas, Rockford, IL, USA) and RNaseOut (20 U; Life Technologies, Carlsbad, CA, USA). The plates with collected cells were immediately placed on dry ice and stored at −80°C until the analysis.

### 2.7 Single-cell gene expression profiling

The collected cells were analyzed by single cell reverse transcription quantitative polymerase chain reaction (scRT-qPCR), to identify changes in the expression of genes that encode membrane proteins participating in astrocyte homeostatic functions or their swelling/volume recovery. In total, the expression of 96 genes was determined. Primers were designed using Primer-BLAST. When possible, each primer pair was separated by at least one intron on the corresponding genomic DNA. For each assay, specificity was tested by melt curve analysis and gel electrophoresis. The effectivity of each assay was determined using a standard dilution over 6 orders of magnitude. In the scRT-qPCR analysis, samples were reverse-transcribed into cDNA using SuperScript III (ThermoFisher Scientific, Waltham, MA, USA). The reverse transcription was performed using the standard protocol recommended by the manufacturer, except for the total volume of 10 μl, an equimolar mix of oligo-dT and random hexamers (50 μM), and a reduced concentration of SuperScript III enzyme (50 U). To monitor the risk of inhibition, RNA TATAA Universal RNA Spike II was added to each reaction, based on the manufacturer’s instructions (TATAA Biocenter, Gpteborg, Sweden). 5 μl of non-diluted cDNA was further pre-amplified using a mix of all primers. Pre-amplified cDNA was diluted 4 times and analyzed using a BioMark instrument (Fluidigm, San Francisco, CA, USA). A detailed description of each procedure is described elsewhere (Rusnakova et al., 2013; Valny et al., 2018).

### 2.8 Data processing

In total, 385 cells from 15 mice were analyzed: 5 Ctrl mice, 4 Aqp4^–/–^ mice, 3 Trpv4^–/–^ mice, and 3 Aqp4^–/–^/Trpv4^–/–^ mice. scRT-qPCR data were pre-processed in Fluidigm Real-Time PCR Analysis software (4.1.2, Fluidigm, San Francisco, CA, USA) and analyzed with GenEx software (Ver. 6.1.1.550, MultiD, Goteborg, Sweden). C_q_ values measured from amplifications that generated melting curves with aberrant Tm were removed, as well as C_q_ values larger than 28. All missing data, for each gene separately, were then replaced with the highest C_q_ +2 (25 % of the lowest measurable concentration). C_q_ values with non-missing data were transformed into relative quantities (scaled to the sample having the lowest expression) and converted to a log2 scale. The data were presented as the mean ± SEM. Statistical analyses of the differences in gene expression among groups were performed using ANOVA for multiple comparisons with Tukey’s *post hoc* test. Differences between the knock-out groups and Ctrl group were considered statistically significant when *p* < 0.01 (Supplementary Table 1).

## 3 Results

Both AQP4 and TRPV4 play an important role during the development of cytotoxic brain edema (Benfenati et al., 2011; Iuso and Križaj, 2016; Pivonkova et al., 2018; Chmelova et al., 2019; Sucha et al., 2022) and they are involved in the process of astrocytic swelling and cell volume regulation. Thus, we suppose that their single- or simultaneous deletion may lead to marked alterations in astrocytic volume and have an impact on brain edema severity and the outcome of ischemic injury. The scRT-qPCR was used to disclose alterations in the expression of genes that are involved in astrocytic swelling and their volume regulation between Ctrl and Aqp4^–/–^, Trpv4^–/–^, and double deficient Aqp4^–/–^/Trpv4^–/–^ mice. In the second part, we performed a comparison of single astrocyte volume changes under exposure to three different ischemia-related pathological stimuli in the cortex of Ctrl and the three knockout groups. Astrocytic volume was quantified based on fluorescence changes in acute brain slices exposed to hypoosmotic stress, extracellular hyperkalemia, and OGD.

### 3.1 Gene expression profiling of single cortical astrocytes isolated from mice lacking AQP4, TRPV4 or both

The scRT-qPCR was conducted to reveal differences in the expression of astrocytic ion channels and transporters, which participate in the maintenance of ionic and neurotransmitter homeostasis and, therefore, under pathological conditions can play a role in the development of brain edema. Cortical EGFP-labeled astrocytes were collected from Ctrl, Aqp4^–/–^, Trpv4^–/–,^ and Aqp4^–/–^/Trpv4^–/–^ mice employing FACS. To ensure we analyzed only astrocytes, we ran a set of pre-tests to exclude cells expressing markers of NG2 glia (chondroitin sulfate proteoglycan 4 – Cspg4, also known as NG2) from our analysis, since NG2 glia are known to express mRNA for GFAP (Leoni et al., 2009). In total, we analyzed a set of 96 genes in 385 astrocytes (127 Ctrl, 113 Aqp4^–/–^, 58 Trpv4^–/–^, 87 Aqp4^–/–^/Trpv4^–/–^; Supplementary Table 1).

**FIGURE 3:**
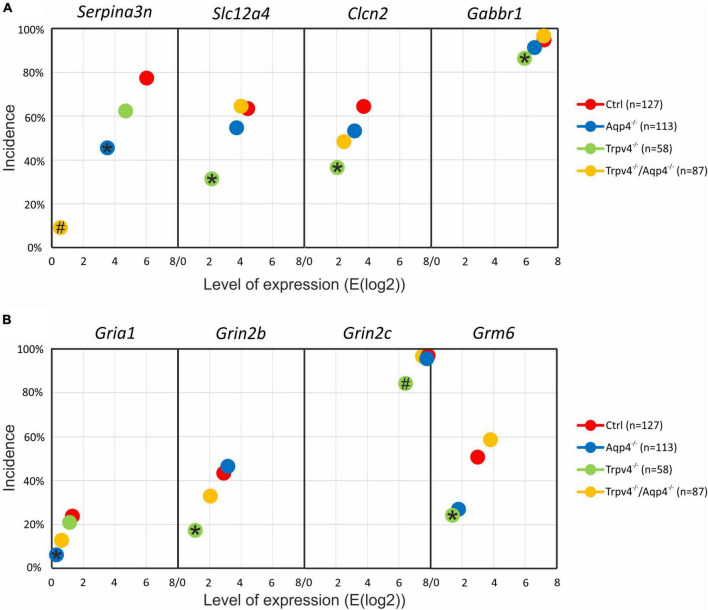
Gene expression profiling. The deletion of AQP4, TRPV4 or both channels caused changes in the expression of several astrocytic genes, such as K^+^ and Cl^–^ ion channels **(A)** or glutamate receptor subunits **(B)**. Note that the highest number of differences was detected in astrocytes from TRPV4 knock-outs, whereas the double knock-out differed from Ctrl in 1 gene only. *Represents a significant difference from Ctrl in the level of expression (*p* < 0.01), and # represents significant differences in the level of expression (*p* < 0.01) together with incidence (*p* < 0.05), data are presented as average values. Aqp4^–/–^, AQP4-deficient; Aqp4^–/–^/Trpv4^–/–^, AQP4- and TRPV4-deficient; Ctrl, control; n, number of cells in each group.

First, we compared the expression levels and numbers of positive cells for mRNA for each gene in our gene set. Using this method, we verified that there are significant differences in the numbers of positive cells for *Aqp4* between our experimental groups. The other gene of interest, *Trpv4*, also showed differences among the groups; however, its expression was only detected in ∼ 6 % of Ctrl cells and 7 % of Aqp4^–/–^ cells. These findings agree with our previous study, where we also found a low expression of *Trpv4* in EGFP-positive astrocytes (Pivonkova et al., 2018). Apart from *Aqp4* and *Trpv4*, we also analyzed the expression of the genes from our preselected gene set and found several differences (*p* < 0.01) between the Ctrl and the rest of our experimental groups (Figure 3).

The deletion of AQP4 channels caused a decrease in the level of mRNA for the Serpina3n (Figure 3A), encoding serine/cysteine peptidase inhibitor subtype a3n (Serpina3n), which is a marker of reactive astrocytes (Zamanian et al., 2012) and its expression increases during inflammation (Takamiya et al., 2002; Xi et al., 2019; Murphy et al., 2020). Similarly, we found a decreased number of cells positive for this gene (about half of the numbers found in the Ctrl group, Figure 3A). We then found a significant decrease in the level of mRNA for the glutamate ionotropic receptor AMPA type subunit 1 as well as in the incidence (*Gria1*, Figure 2B). The deletion of TRPV4 channels led to a significant decrease in the expression of *Slc12a4* encoding KCC1 cotransporter together with *Clcn2* (gene for ClC2 channel), *Gabbr2* (encoding gamma-aminobutyric acid (GABA) B receptor 2; GABA_B_), *Grin2b* and *Grin2c* (encoding glutamate ionotropic receptor NMDA type subunit 2B and 2C), and *Grm6* (encoding metabotropic glutamate receptor 6; mGluR6), compared to the Ctrl group. Moreover, with all these genes we also saw a decrease in the incidence of positive cells, however, the differences were not significant, except for the *Grin2c* (Figures 3A, B). Finally, the simultaneous deletion of both channels led to a decrease in the level of *Serpina3n* expression as well as in the incidence of astrocytes expressing this gene. Interestingly, astrocytic *Serpina3n* levels were decreased to a minimum in Aqp4^–/–^/ Trpv4^–/–^ mice when compared to Ctrl.

Considering the gene expression of ion channels and transporters that participate in astrocytic homeostatic functions and cell swelling/cell volume regulation, we found rather minor differences between the knock-out groups and Ctrl. The highest number of differences were shown in astrocytes from Trpv4^–/–^ mice, whereas the lowest number was detected in the mice lacking both AQP4 and TRPV4 channels. The expression profile of astrocytes from Aqp4^–/–^ mice was similar to that observed in Ctrl and Aqp4^–/–^/Trpv4^–/–^ mice.

### 3.2 The effect of AQP4 and TRPV4 deletion on the swelling of astrocytes and their volume recovery in response to pathological stimuli

To investigate the role of AQP4 and TRPV4 channels in the astrocyte swelling and their volume regulation, astrocytic volume changes evoked by exposure to various pathological stimuli were quantified in the cortex of AQP4-, TRPV4- and double knockout mice with EGFP-labeled astrocytes and compared to those observed in the Ctrl. The astrocytic volume changes were evoked by three different solutions: H-100, 50 mM K^+^ and OGD. The changes in the volume of astrocyte soma were recorded every 10 min during a 20-min application of solution mimicking pathological stimulus, as well as during the subsequent 20-min washout in iso-osmotic aCSF (Figure 1).

**FIGURE 4:**
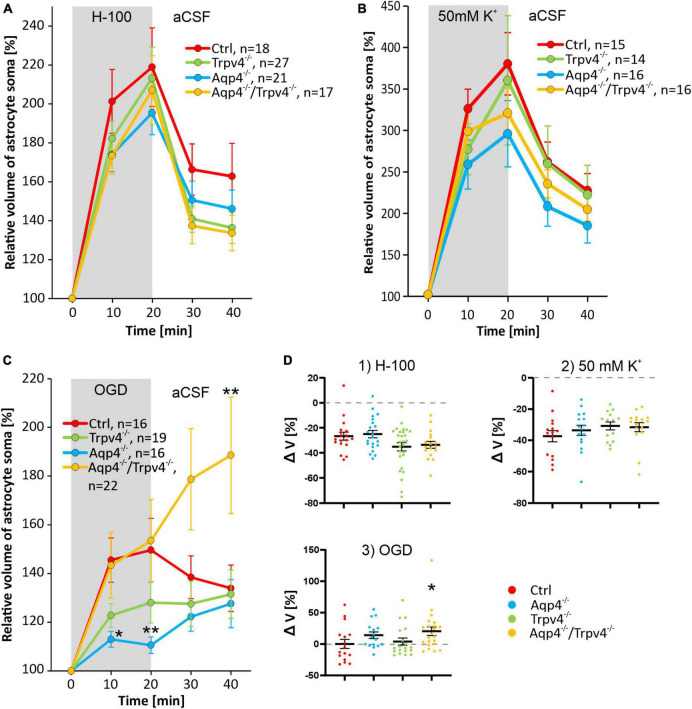
Pathologically induced swelling of cortical astrocyte soma - the role of AQP4 and TRPV4. **(A)** Time course of the volume changes of cortical astrocyte soma in response to 20 min application of 200 mOsmol hypotonic solution (H-100, gray area) followed by 20 min of washout in artificial cerebrospinal fluid (aCSF). **(B)** Time course of volume changes of cortical astrocyte soma after 20 min exposure to hyperkalemia (50 mM K^+^, gray area) followed by 20 min washout. **(C)** The effect of 20 min of oxygen-glucose deprivation (OGD, gray area) on astrocyte soma volume. The OGD exposure was followed by 20 min of washout. **(D)** Average volume recovery (ΔV) during the washout. Note that the exposure to hypoosmotic stress or to hyperkalemia did not cause differences in the swelling of astrocyte soma from AQP4-deficient (Aqp4^–/–^), TRPV4-deficient (Trpv4^–/–^) or double-deficient (Aqp4^–/–^/Trpv4^–/–^) mice, when compared to controls (Ctrl). On the contrary, OGD caused a significant decrease in the volume of the soma of Aqp4^–/–^ astrocytes (*p < 0.05, **p < 0.01). Moreover, we detected a significant increase in the volume of soma of Aqp4^–/–^/Trpv4^–/–^ astrocytes after 20 min of washout, compared to Ctrl, which indicates impaired volume recovery, shown in panel D3 (*p < 0.05). Data are presented as mean ± SEM; n, number of cells in each group.

After 20 min of H-100 application, we found a significant volume increase in all four experimental groups, on average the astrocyte soma reached a volume of 218.93 ± 20.19 % in Ctrl mice (*n* = 18), 195.41 ± 11.20 % in Aqp4^–/–^ mice (*n* = 21), 213.31 ± 15.81 % in Trpv4^–/–^ mice (*n* = 27) and 207.08 ± 17.67 % in Aqp4^–/–^/Trpv4^–/–^ mice (*n* = 17; Figure 4A). Interestingly, under these conditions, we did not find any differences between the astrocyte volume changes of the Ctrl and Aqp4^–/–^, Trpv4^–/–^ and Aqp4^–/–^/Trpv4^–/–^ mice. Next, we quantified the volume changes in the astrocyte soma exposed to hyperkalemia, which induced a significant increase in the astrocytic volume. After the 20 min of 50 mM K^+^ application, the astrocytic volume reached the following values: 380.36 ± 37.73 % (*n* = 15) in Ctrl, 296.02 ± 39.98 % (*n* = 16) in Aqp4^–/–^, 360.00 ± 77.90 % (*n* = 14) in Trpv4^–/–^, 320.89 ± 29.98 % (*n* = 16) in Aqp4^–/–^/Trpv4^–/–^ (Figure 4B). Similar to the results obtained during hypoosmotic stress, we did not observe any significant differences between the volume changes in astrocytes from Ctrl and the rest of our experimental groups. Moreover, volume changes of the astrocyte soma were quantified during and after the exposure to OGD (Figure 4C). In astrocytes from the Ctrl mice, we observed an increase in the somatic volume from 100 % to 149.66 ± 13.02 % after 20 min of OGD (*n* = 16), and only a moderate volume recovery during the washout (133.94 ± 9.51 % after 20 min of washout; *n* = 16). The volume changes of astrocytes from Trpv4^–/–^ mice did not differ significantly from the Ctrl group at any time point (135.75 ± 9.42 % after 20 min of OGD and 131.58 ± 9.95 % after 20 min of washout; *n* = 19). On the contrary, the astrocytes from Aqp4^–/–^ mice reached a significantly lower volume during OGD (110.58 ± 3.38 % after 20 min of OGD; *n* = 16), whereas the astrocytes from Aqp4^–/–^/Trpv4^–/–^ mice swelled similarly to the Ctrl during OGD but reached a significantly higher volume after 20 min of washout (153.51 ± 16.92 % after 20 min of OGD and 188.54 ± 23.86 % after 20 min of washout; *n* = 22). Finally, the volume recovery during washout was quantified as the difference between the volume after 20 min of pathological solution and the volume after 20 min of washout (Figure 4D). We did not detect any differences in the volume recovery between the experimental groups after application of either H-100 or 50 mM K^+^. However, after OGD application the astrocytes from Aqp4^–/–^/Trpv4^–/–^ mice showed impaired volume recovery. Their volume increased by 20.3 ± 6.7 %, compared to Ctrl (volume decrease of 2.8 ± 7.4 %; Figure 4D). For all individual data and statistical values see Supplementary Figures 1–3 and Supplementary Table 2. Overall, the lack of AQP4, TRPV4, or even of both channels, had no effect on the ability of astrocytes to swell or to recover their volume when exposed to hypoosmotic stress or hyperkalemia. However, during astrocyte exposure to OGD the lack of AQP4 resulted in significantly reduced swelling of astrocyte soma, and the lack of both channels, AQP4 and TRPV4, led to the disruption of astrocyte volume recovery following OGD. The lack of TRPV4 appeared to result in slower swelling of astrocyte soma during the OGD.

As we detected a marked variability in the astrocytic volume changes, even in response to individual pathological stimuli, we performed Kohonen’s self-organizing maps (SOM) and principal component analysis (PCA), to determine whether there are distinct astrocytic subpopulations with a different ability to swell or regulate their volume as already described by Benesova et al. (2009, 2012). Contrary to their analysis, the SOM and PCA enable taking the entire time-course of swelling into account, as well as the volume recovery during washout in individual astrocytes, and therefore do not require any pre-determined threshold to divide the cells into subpopulations. First, we analyzed the volume changes of astrocytes in the cortex of the Ctrl mice. The obtained parameters of two distinct subpopulations were then applied to those lacking AQP4, TRPV4, or both channels.

### 3.3 The impact of the deletion of AQP4 and/or TRPV4 channels on swelling/volume regulation of two astrocytic subpopulations

**FIGURE 5:**
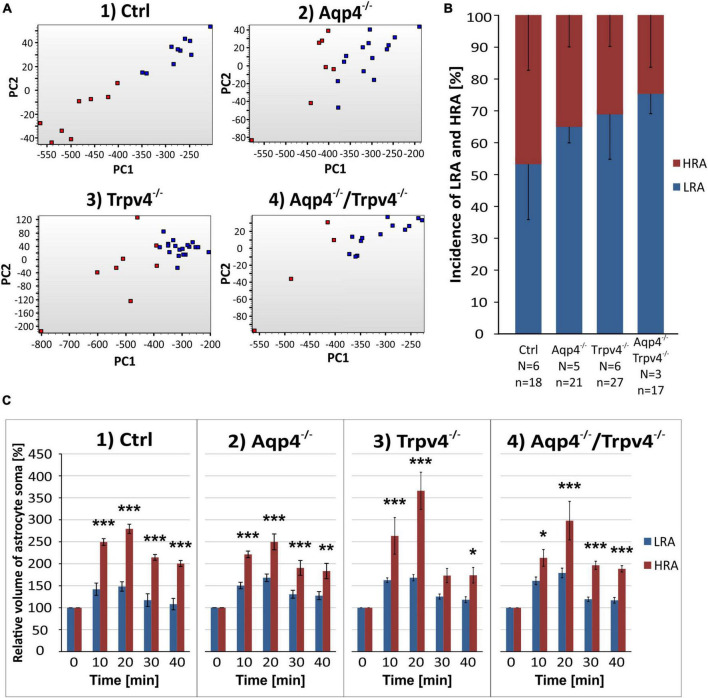
Identification of two astrocytic populations with different volume responses after hypoosmotic stress. **(A)** Principal component analysis of astrocytic volume changes evoked by 200 mOsmol hypotonic solution. We determined two populations (HR, red and LR, blue) of astrocytes in the control group (A.1) and then applied the same parameters to the rest of our experimental groups (A.2-4). **(B)** Incidence of LR and HR astrocytes in our experimental groups counted as a percentage of all the astrocytes measured per experimental animal. **(C)** Relative volume changes of the soma of LR and HR astrocytes after exposure to hypoosmotic stress within our experimental groups. The volume of HR astrocytes in each group was significantly higher than that of LR astrocytes: *p < 0.05, **p < 0.01, ***p < 0.001. Data are presented as mean ± SEM. Aqp4^–/–^, AQP4-deficient; Aqp4^–/–^/Trpv4^–/–^, AQP4- and TRPV4-deficient; Ctrl, control; HR, high-responding; LR, low-responding; N, number of animals used in each group; n, number of cells (HR + LR astrocytes); PC, principal component; Trpv4^–/–^, TRPV4-deficient.

**FIGURE 6:**
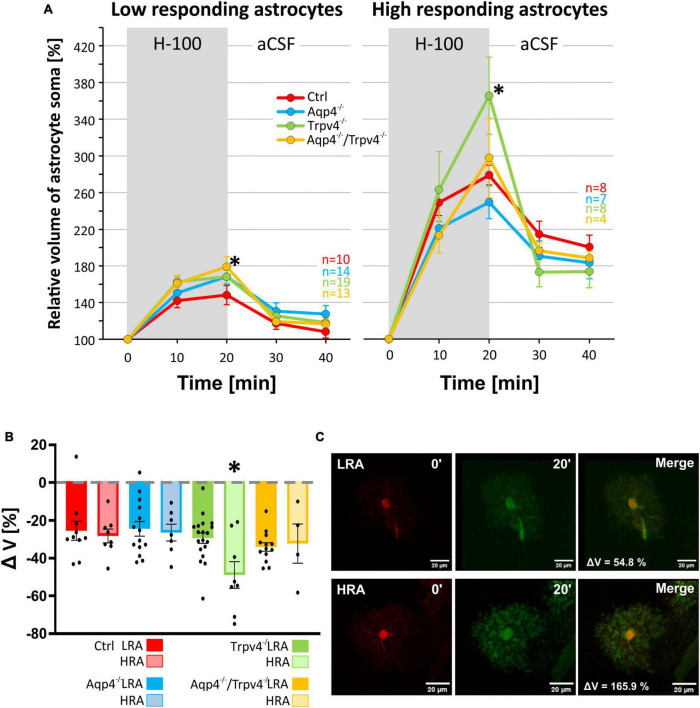
Differences in volume of low-responding (LR) and high-responding (HR) astrocytic subpopulations after exposure to hypoosmotic stress. **(A)** The time course of volume response of the soma of LR and HR cortical astrocytes to the exposure to hypoosmotic stress (H-100, gray area) followed by 20 min of washout. Note that the LR astrocytes only from Aqp4^–/–^/Trpv4^–/–^ animals differed significantly from Ctrl (*p < 0.05) after 20 min of H-100. In the HR population of astrocytes only the volume of Trpv4^–/–^ cells differed significantly from Ctrl group after 20 min of H-100 application. **(B)** Volume recovery (ΔV) of LR and HR astrocytes counted as the difference between the maximal volume after 20 min of H-100 application, and the final volume after 20 min of washout. Only HR astrocytes from Trpv4^–/–^ mice differed from the appropriate Ctrl subpopulation. **(C)** Representative images of the cells (both from Ctrl strain) prior to (0′), and during (20′) application of the H-100. Data are presented as mean ± SEM. aCSF, artificial cerebrospinal fluid; Aqp4^–/–^, AQP4-deficient; Aqp4^–/–^/Trpv4^–/–^, AQP4- and TRPV4-deficient; Ctrl, control; H-100, 200 mOsmol hypotonic solution; HR, high-responding; LR, low-responding; n, number of cells; Trpv4^–/–^, TRPV4-deficient.

We subjected the astrocyte volume changes induced by three different pathological stimuli, to SOM and PCA analysis. Based on the volume increase detected in the cell soma in response to the hypoosmotic stress, and subsequent volume decrease during washout, we clearly identified two different subpopulations of astrocytes in the cortex of the Ctrl mice (*n* = 18 cells; Figure 5A) which we further termed low-responding astrocytes (LRA) and high-responding astrocytes (HRA). When compared to LRA, the relative volume changes of the soma of HRA in Ctrl mice were almost doubled during the application of H-100, as well as during washout (Figure 5C). Based on the parameters defined in the Ctrl group, LRA and HRA were also identified in Aqp4^–/–^, Trpv4^–/–^ and Aqp4^–/–^/Trpv4^–/–^ mice (Figures 5A, C). The issue then arose as to whether the incidence of individual subpopulations, quantified as a percentage of LRA and HRA in each animal, differs between our experimental groups (Figure 5B). Nevertheless, we found no differences in the incidence of LRA and HRA between the groups. Furthermore, swelling of LRA in Aqp4^–/–^ and Trpv4^–/–^ mice was comparable to that observed in the Ctrl mice. Similarly, we detected no differences in their ability to restore their volume during washout (counted as a difference between the maximal swelling 20 min after H-100 application and final volume after 20 min of washout; Figures 6A–C). However, the deletion of both channels in Aqp4^–/–^/Trpv4^–/–^ mice led to a significantly higher swelling of LRA after 20 min of H-100 application (148.22 ± 10.46 % in Ctrl; 179.09 ± 10.92 % in Aqp4^–/–^/Trpv4^–/–^ mice; Figure 6A), but this difference did not affect their volume recovery during washout (Figure 6B). The swelling of HRA and their volume recovery during washout in Aqp4^–/–^ and Aqp4^–/–^/Trpv4^–/–^ mice were comparable to those observed in the Ctrl mice (Figure 6B). However, the swelling of HRA from Trpv4^–/–^ mice was significantly increased after 20 min of hypoosmotic stress (366.08 ± 42.32 %), when compared to Ctrl HRA (279.40 ± 10.78 %; Figure 6A). In addition, we found more efficient volume recovery in astrocytes from Trpv4^–/–^ mice (−48.91 ± 7.10 %) compared to Ctrl (−28.22 ± 3.64 %; Figure 6B). For individual data and details of the statistics see Supplementary Figures 4, 5 and Supplementary Tables 3, 4.

**FIGURE 7:**
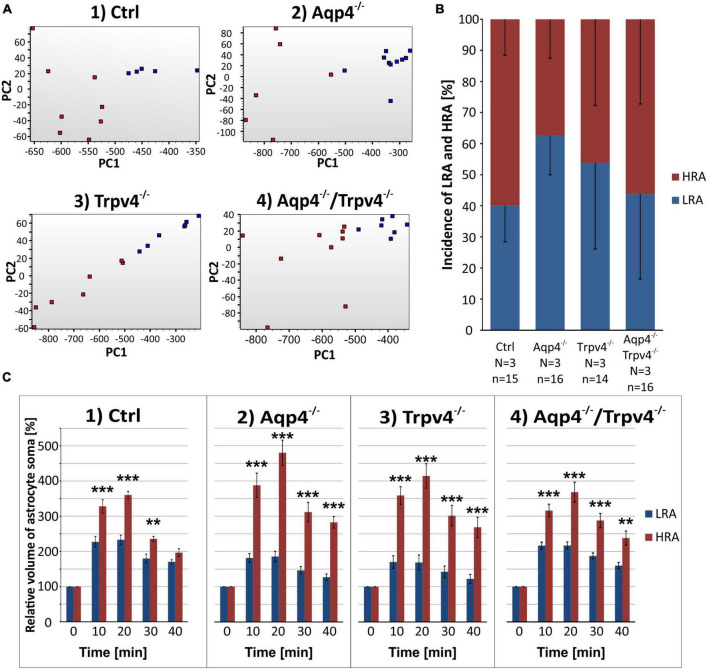
Identification of two astrocytic subpopulations with different volume responses to hyperkalemia. **(A)** Principal component analysis of the volume changes in astrocyte soma evoked by the exposure to hyperkalemia. We determined two subpopulations (HR, red and LR, blue) of astrocytes in Ctrl group (A.1) and then applied the same parameters to the rest of our experimental groups (A.2-4). **(B)** Incidence of LR and HR astrocytes in our experimental groups counted as a percentage of all the astrocytes measured in one experimental animal. **(C)** Relative volume changes of the soma of LR and HR astrocytes after the exposure to hyperkalemia within our experimental groups. The volume of HR astrocytes in each group was significantly higher than that of LR astrocytes: **p < 0.01, ***p < 0.001. Data are presented as mean ± SEM. Aqp4^–/–^, AQP4-deficient; Aqp4^–/–^/Trpv4^–/–^, AQP4- and TRPV4-deficient; Ctrl, control; HR, high responding; LR, low responding; N, number of animals used in each group; n, number of cells (HR + LR astrocytes); PC, principal component; Trpv4^–/–^, TRPV4-deficient.

**FIGURE 8:**
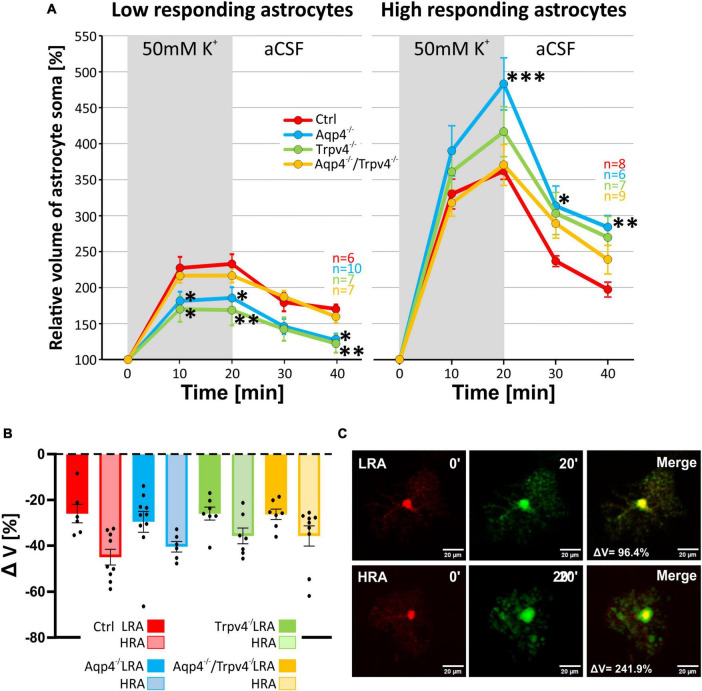
Differences in the volume of low-responding (LR) and high-responding (HR) astrocytic populations after the exposure to hyperkalemia. **(A)** The time course of volume response of the soma of LR and HR cortical astrocytes to the exposure to 50 mM K^+^ (gray area) followed by 20 min of washout. The LR astrocytes from Aqp4^–/–^ and from Trpv4^–/–^ mice reached significantly smaller volume (*p < 0.05; **p < 0.01) after 20 min of 50 mM K^+^ as well as after 20 min of washout, compared to Ctrl. In the HR population of astrocytes only the volume of Aqp4^–/–^ cells differed significantly from Ctrl group (*p < 0.05; **p < 0.01; ***p < 0.001) after 20 min of 50 mM K^+^ application and during the washout. **(B)** Volume recovery (ΔV) of LR and HR astrocytes counted as difference between the maximal volume after 20 min of 50 mM K^+^ application, and final volume after 20 min of washout. **(C)** Representative images of the cells prior to (0′), and during (20′) the application of 50 mM K^+^. The representative LR cell belongs to the Aqp4^–/–^ strain, and the HR cell is from the Ctrl strain. Data are presented as mean ± SEM. aCSF, artificial cerebrospinal fluid; Aqp4^–/–^, AQP4-deficient; Aqp4^–/–^/Trpv4^–/–^, AQP4- and TRPV4-deficient; Ctrl, control; HR, high responding; LR, low responding; n, number of cells; Trpv4^–/–^, TRPV4-deficient; 50 mM K^+^, 50 mM hyperkalemic solution.

Likewise, the statistical analysis of the variability in the volumetric responses to hyperkalemia also led to the identification of two subpopulations of astrocytes in all four experimental groups (Figures 7A–C). When we analyzed the incidence of LRA and HRA in the experimental groups, we did not detect any differences (Figure 7B). We then compared the volume changes of LRA from all four groups after 10 and 20 min of 50 mM K^+^ application (Figures 8A–C). This comparison revealed that the LRA from Aqp4^–/–^ and Trpv4^–/–^ mice swelled less than LRA from Ctrl mice after 10 and 20 min of 50 mM K^+^ application (Aqp4^–/–^: 185.64 ± 14.96 %, n_LR_ = 10; Trpv4^–/–^: 168.82 ± 21.28 %, n_LR_ = 7; Ctrl: 233.00 ± 13.32 %; n_LR_ = 7 after 20 min of 50 mM K^+^ application). Moreover, both groups also reached a significantly smaller volume after 20 min of washout. Interestingly, the LRA from Aqp4^–/–^/Trpv4^–/–^ mice did not differ from the Ctrl LRA at any time point (Figure 8A). On the contrary, when we compared the volumes of the HRA subpopulations, we found a significant increase in the volume of HRA from Aqp4^–/–^ mice after 20 min of 50 mM K^+^ application (Aqp4^–/–^: 479.99 ± 35.86 %, n_HR_ = 6; Ctrl: 360.01 ± 11.57 %, n_HR_ = 8) and after 10 and 20 min of washout (Aqp4^–/–^: 282.58 ± 16.22 %; Ctrl: 196.57 ± 11.04 % after 20 min of washout; Figure 8A). When we compared the volume recovery of Trpv4^–/–^ and double knockout groups to the appropriate Ctrl subpopulation, we did not see any differences (Figure 8B). All the individual data and detailed statistical values can be found in the Supplementary Figures 6, 7 and in the Supplementary Table 3, resp. 4. Overall, the lack of AQP4 channels had the opposite effects on volume changes in the LRA and HRA subpopulations, the lack of TRPV4 channels affected only the LRA, resulting in lesser volume changes in response to hyperkalemia. It is noteworthy that the deletion of both channels together did not have any effect on either LRA or HRA under exposure to hyperkalemia.

**FIGURE 9:**
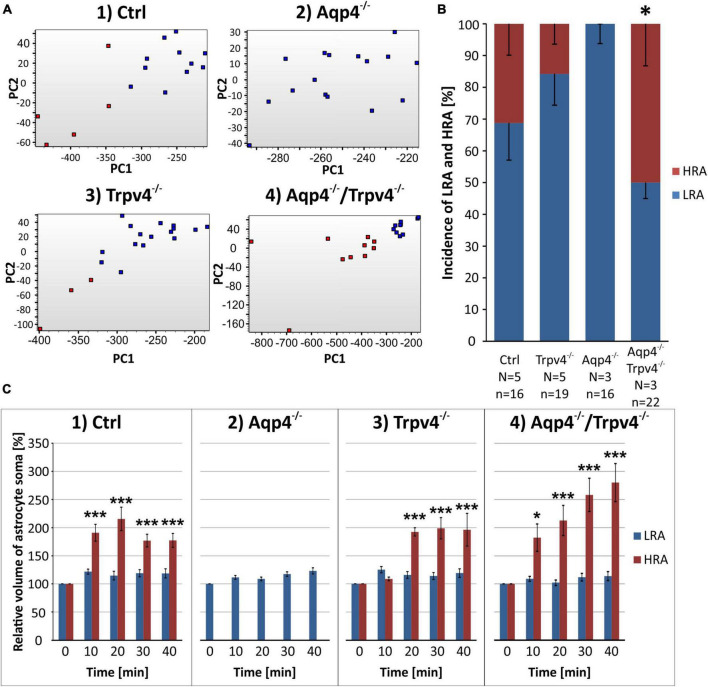
Identification of two astrocytic populations with different volume responses to OGD. **(A)** Principal component analysis of the volume changes in the soma of cortical astrocytes evoked by the OGD. We determined two populations (HR, red and LR, blue) of astrocytes in Ctrl group (A.1) and then the same parameters were applied to the rest of the experimental groups (A.2-4). **(B)** Incidence of LR and HR astrocytes in our experimental groups counted as a percentage of all the astrocytes measured in one experimental animal. **(C)** Relative volume changes of the soma of LR and HR astrocytes after the OGD within our experimental groups (C.1-4). Interestingly, we were unable to detect HR astrocytes in the Aqp4^–/–^ mice. Data are presented as mean ± SEM; results were considered significant when *p < 0.05, ***p < 0.001. Aqp4^–/–^, AQP4-deficient; Aqp4^–/–^/Trpv4^–/–^, AQP4- and TRPV4-deficient; Ctrl, control; HR, high-responding; LR, low-responding; N, number of animals used in each group; n, number of cells (HR + LR astrocytes); OGD, oxygen-glucose deprivation; PC, principal component; Trpv4^–/–^, TRPV4-deficient.

**FIGURE 10:**
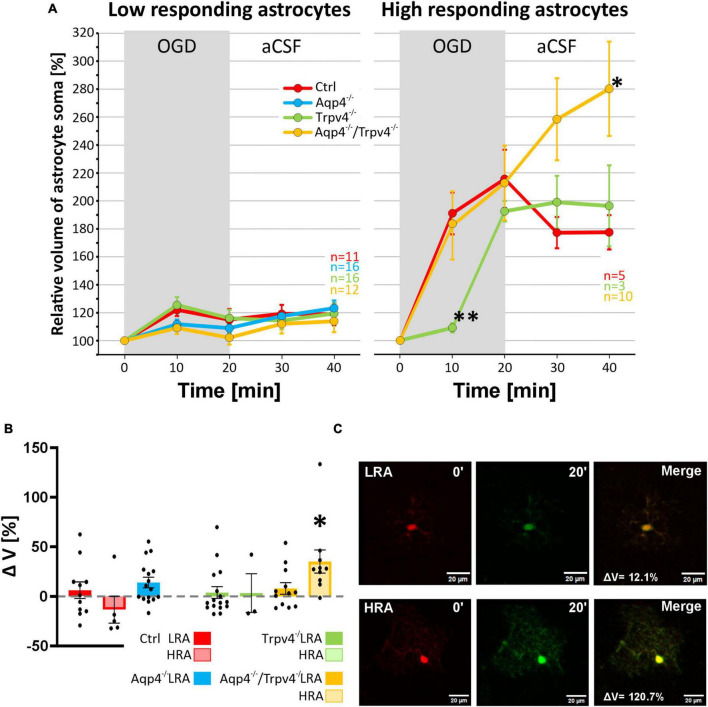
Differences in the volume of low-responding (LR) and high-responding (HR) astrocytic populations after OGD. **(A)** The time course of volume response of the soma of LR and HR cortical astrocytes to the OGD (gray area) followed by 20 min of washout. Note that HR astrocytes from Trpv4^–/–^ mice differed significantly from Ctrl group (**p < 0.01) after 10 min of OGD and the Aqp4^–/–^/Trpv4^–/–^ astrocytes reached significantly higher volume than Ctrl after 20 min of washout (*p < 0.05). **(B)** Volume recovery (ΔV) of LR and HR astrocytes counted as difference between the maximal volume after 20 min of OGD, and the final volume after 20 min of washout. HR astrocytes from Aqp4^–/–^/Trpv4^–/–^ mice failed to recover their volume during washout (*p < 0.05). **(C)** Representative images of the cells prior to (0′) and during (20′) OGD. The LR astrocyte belongs to the Aqp4^–/–^ group, and the HR astrocyte is from the Aqp4^–/–^/Trpv4^–/–^ group. Data are presented as mean ± SEM. Aqp4^–/–^, AQP4-deficient; Aqp4^–/–^/Trpv4^–/–^, AQP4- and TRPV4-deficient; Ctrl, control; n, number of cells; OGD, oxygen-glucose deprivation; Trpv4^–/–^, TRPV4-deficient.

Finally, data representing the volume changes of astrocytes exposed to OGD were also analyzed for the existence of different astrocyte subpopulations. Consistently with the data described above, we also detected LRA and HRA in the cortex of the Ctrl mice with significantly different volume responses to the OGD (Figures 9A–C). We detected HRA and LRA in the Trpv4^–/–^ (n_LR_ = 16, n_HR_ = 3) and Aqp4^–/–^/Trpv4^–/–^ (n_LR_ = 12, n_HR_ = 10) groups but we were unable to detect any HRA within the Aqp4^–/–^ mice and, consequently, all the measured cells were categorized as LRA (Figure 9). Comparing the incidence of LRA and HRA in Ctrl, Trpv4^–/–^ and Aqp4^–/–^/Trpv4^–/–^ mice, we found a significant increase in the number of HRA within the cortex of Aqp4^–/–^/Trpv4^–/–^ mice (31.25 ± 9.88 % of HRA in Ctrl to 50.00 ± 13.21 % of HRA in Aqp4^–/–^/Trpv4^–/–^; Figure 9B). The volume responses of LRA from all experimental groups were similar regardless of the presence/absence of AQP4 and TRPV4 channels (Figures 10A–C). On the contrary, we found significant differences between the volume responses of HRA. When compared to the Ctrl, the volume of HRA from Trpv4^–/–^ mice was significantly lower after 10 min of OGD (Ctrl: 191.00 ± 15.02 %, Trpv4^–/–^: 109.05 ± 3.25 %), however, the volume of Trpv4^–/–^ and Aqp4^–/–^/Trpv4^–/–^ after 20 min of OGD was comparable (Figure 10A). Contrary to the Trpv4^–/–^, the HRA from Aqp4^–/–^/Trpv4^–/–^ mice swelled similarly to the Ctrl during the OGD. However, their volume did not recover during washout and even continued to rise (280.16 ± 33.76 %), and after 20 min it was significantly higher than the volume of the Ctrl (177.42 ± 12.31 %; Figure 10A). Accordingly, when we compared the volume recovery during washout, we found no volume recovery in the HRA from Aqp4^–/–^/Trpv4^–/–^ mice but instead we detected a further volume increase (35.12 ± 11.68 % in Aqp4^–/–^/Trpv4^–/–^ mice; −13.38 ± 13.59 % in Ctrl; Figure 10B). We thus summarize that under the conditions of OGD the lack of AQP4 and/or TRPV4 channels does not affect the volume of LRA. On the contrary, the HRA are strongly affected. The deletion of AQP4 channels prevents HRA from increasing their volume, so it is comparable to the LRA. The lack of both AQP4 and TRPV4 channels resulted in the disability of the HRA subpopulation to restore their volume during washout following OGD, whereas the lack of TRPV4 channels led to a delay in HRA swelling.

## 4 Discussion

We herein present a study of the effect of TRPV4 and AQP4 channel deletion on astrocyte swelling and their ability to regulate cell volume under conditions modeling cerebral ischemia. In addition, we examined whether the deletion of TRPV4 and AQP4 could affect the expression of other ion channels and transporters involved in astrocyte swelling and their volume regulation. Various *in vitro* and *in vivo* studies (Benfenati et al., 2011; Jo et al., 2015; Iuso and Križaj, 2016; Salman et al., 2017; Chmelova et al., 2019; Sucha et al., 2022) have shown that these two channels can form functional complexes and jointly participate in the development of cytotoxic edema in ischemic brain injury, but the results of these experiments are not fully consistent. Since TRPV4 also interacts with other ion channels, such as K^+^ channels (Liu et al., 2020a,b), we expected that deletion of TRPV4 might also affect their expression and/or localization.

When we evaluated the changes in cell volume triggered by ischemia-modeling conditions of all measured astrocytes together, we observed no significant differences in either AQP4, TRPV4 or double knockouts when compared to Ctrl. We could only spot changes under OGD conditions in which we detected a decrease in astrocyte volume in AQP4 knockouts and reduced volume recovery in double knockouts compared to Ctrl. However, when we grouped astrocytes according to how much they changed their volume in response to the application of ischemia-modeling solutions, we got two subpopulations called HRA and LRA. Such division has been described previously in Benesova et al. (2009), but the methodological approach was different in our case. While Benesova et al. (2009) divided the cells based on the determination of a threshold of 10% volume increase, we used PCA analysis which takes into account not only absolute values but also the overall pattern of volume changes over time. In addition, we uncovered changes in the expression of some astrocytic receptors, channels, and transporters that result from the deletion of the AQP4 or TRPV4 and may also affect the volume changes of astrocytes under ischemic conditions.

### 4.1 AQP4 is not essential for astrocyte swelling during hypoosmotic stress but contributes significantly to the swelling induced by oxygen-glucose deprivation

Aquaporin 4 represents the main water channel in the cell membrane of astrocytes, but the passage of water through this channel is not the only mechanism by which water enters cells. It has been clearly demonstrated that under hypoosmotic conditions astrocytes markedly take up water and swell, however, this swelling is purely osmotic and therefore passive in nature (Reinehr et al., 2007; Hirrlinger et al., 2008; Nase et al., 2008; Stokum et al., 2016). This volume increase triggers a process of volume regulation in which osmolytes, and water are released into the ECS and in which AQP4 and TRPV4 channels are suspected to play a crucial role (Benfenati et al., 2011). In addition to AQP4, water can enter astrocytes via several routes, such as diffusion, co-transport with ions, or paracellularly through astrocytic syncytium (Stokum et al., 2015).

Our results showed that deletion of AQP4 does not affect astrocyte swelling under hypoosmotic stress. This result is not surprising, because it agrees with the results of Murphy et al. (2017), Barile et al. (2023), who reported that the swelling of astrocytes does not require AQP4. Similarly, Mola et al. (2016) showed that cells lacking AQP4 can swell, albeit more slowly, but are unable to trigger the RVD. In general, astrocyte membranes are quite highly permeable for water (Przybyło et al., 2021), therefore a strong osmotic challenge, such as our application of H-100 solution, is most likely sufficient to create strong osmotic gradients, resulting in the whole astrocyte swelling even without the involvement of AQP4 channels (Walch and Fiacco, 2022). However, AQP4 channels seem to be involved in the removal of water from astrocyte and in their volume recovery, which also likely explains the worsening of cerebral edema in Aqp4^–/–^ mice in the experimental models of subarachnoid hemorrhage or permanent middle cerebral artery occlusion (MCAO) (Manley et al., 2004; Tait et al., 2010).

It is also important to note that under physiological conditions, astrocytic expression of AQP4 predominates on perivascular and subpial processes, where it is responsible for water exchange between the neuropil and the cerebral bloodstream or cerebrospinal fluid (Nielsen et al., 1997; Rash et al., 1998). This could be the reason why we do not observe an effect of AQP4 deletion on astrocyte swelling in our experiments, where we only determine cell soma volume. However, our previous study using α-syntrophin-negative mice (which show a marked loss of AQP4 from perivascular and subpial membranes but no decrease in other membrane domains, (Amiry-Moghaddam et al., 2003) has shown that the astrocytic soma swells regardless of AQP4 localization (Anderova et al., 2014). This suggests that in acute tissue slices deprived of blood flow and blood pressure, water is transported to astrocytes throughout the entire membrane and not just by endfeet, and other transport mechanisms are likely involved.

The most prominent effect of AQP4 deletion was detected during the OGD. The application of OGD in our experiments revealed differences in astrocytic swelling regardless of the categorization of the cells into LRA or HRA groups. We observed significantly decreased swelling of astrocytes with deleted AQP4 when compared to the Ctrl. This finding agrees with numerous studies that reported improved conditions of experimental animals with downregulated/deleted/inhibited AQP4, after the induction of cerebral ischemia (Manley et al., 2000; Li et al., 2015; Yang et al., 2015; Liu et al., 2017). The decreased volume of astrocytes from Aqp4^–/–^ mice under exposure to OGD indicates an impairment of compensatory mechanisms that allow water influx into astrocytes, which we detected in the other models (hypoosmotic stress and hyperkalemia). One of these mechanisms may be represented by connexin hemichannels. From this protein family, connexin-43 (Cx43) is predominantly expressed in astrocytes, and it was described to functionally interact with AQP4 channels (Nicchia et al., 2005; Li et al., 2015). In healthy tissue, Cx43 represents a water pathway that can compensate for the lack of AQP4 channels. However, cerebral ischemia in the form of experimental OGD causes changes in the phosphorylation state of Cx43 (Le et al., 2014) and subsequently decreases the protein levels and leads to the relocation of Cx43 from the plasmatic membrane into the cytoplasm (Wu et al., 2015). The absence of this compensatory mechanism seems to reveal the effect of AQP4 deletion that was masked by above mentioned mechanisms. Therefore, we can say that deletion of AQP4 causes impairment of water transport in astrocytes and leads to a decrease in cell swelling. It explains why we detected only a negligible volume increase in astrocytes from Aqp4^–/–^ mice during OGD and we were unable to detect any HRA.

Interestingly, using the scRT-qPCR method we detected decreased levels of Serpina3n in the astrocytes of AQP4 knockouts. This acute-phase protease inhibitor is secreted in response to inflammation and is considered a potential marker of reactive astrogliosis. Its role in the CNS is not entirely clear. On one hand, as a protease inhibitor, it acts protectively in neuropathic pain or multiple sclerosis, on the other hand, its increased amount aggravates neuroinflammation and neurotoxicity (Vicuña et al., 2015; Liu et al., 2023). Recent work has shown that it plays a neuroprotective role in ischemic brain injury and that its increased expression leads to improved outcomes after transient MCAO, possibly via interacting with clusterin and inhibiting neuronal apoptosis and neuroinflammation (Zhang et al., 2022). Although SERPINa3n expression does not seem to have a direct effect on the volume changes of astrocytes, its reduced expression in AQP4 knockouts could contribute to the exacerbation of ischemic injury, as we have shown in our previous work (Sucha et al., 2022).

### 4.2 The deletion of AQP4 has different effects on HRA and LRA volume changes during exposure to high potassium concentrations

Surprisingly, we detected opposing effects of AQP4 deletion on volume changes in LRA and HRA during hyperkalemia, which was hidden during the initial analysis that included all cells together. While deletion of AQP4 caused a reduction of hyperkalemia-induced swelling in LRA, the swelling was higher in HRA, compared to Ctrl subpopulations. It was demonstrated that AQP4 channels do not play a role in the K^+^-induced astrocytic swelling (Rosic et al., 2019; Walch et al., 2020; Toft-Bertelsen et al., 2021) and that the water flows into the cells via different channels, such as connexins, pannexins or cotransporters (Strohschein et al., 2011; Scemes and Spray, 2012), or directly across the plasmatic membrane (Walch and Fiacco, 2022). Despite the fact that several studies have suggested interactions between AQP4 channels and Na^+^/K^+^ ATPase in the clearance of extracellular K^+^ (Illarionova et al., 2010; Strohschein et al., 2011), Walch et al. (2020) showed that even though the Na^+^/K^+^ ATPase comprises significant part of astrocyte swelling during mild or moderate hyperkalemia, its action is AQP4 independent. The uptake of K^+^ causes water influx into the astrocytes and their consequent swelling, which is proportional to the astrocytic ability to buffer K^+^ (Neprasova et al., 2007; Risher et al., 2009; Kolenicova et al., 2020). It is therefore possible that the low and high response of astrocytes to hyperkalemia reflects their ability to buffer extracellular K^+^. However, our results from Aqp4^–/–^ astrocytes did not show any changes in the levels of Na^+^/K^+^ ATPase or even NKCC or KCC transporters, which are the first suspects associated with the clearance of excessive K^+^ from the ECS, especially in high extracellular concentration of K^+^ ([K^+^]_O_) (Murakami and Kurachi, 2016; Walch and Fiacco, 2022). Walch et al. (2020) suggested that the role of AQP4 in the volumetric response of astrocytes induced by hyperkalemia is in the water efflux from the cells. It limits the astrocytic swelling and thus its deletion leads to an increase in the astrocyte volume (Walch et al., 2020) the same as we detected in HRA from Aqp4^–/–^ mice. On the contrary, we detected a decreased volume of LRA from the Aqp4^–/–^ mice, indicating involvement of other factors such as location/contacts with other cell types.

### 4.3 TRPV4 deletion mainly affects the swelling of HRA under hypoosmotic stress and oxygen-glucose deprivation

As already mentioned, astrocyte swelling triggers the mechanisms responsible for the transport of osmolytes and water out of the cell, resulting in RVD. These mechanisms include the opening of the TRPV4 channel, followed by Ca^2+^ influx and activation of Ca^2+^-dependent K^+^ channels or VRACs (Benfenati et al., 2011; Jo et al., 2015; Tureckova et al., 2023). Although this mechanism has been demonstrated *in vitro* (Benfenati et al., 2011; Jo et al., 2015; Iuso and Križaj, 2016), it has not been confirmed in acute brain slices.

In this study, when the volumetric changes of astrocytes were compared together, regardless of the existence of HRA and LRA groups, we observed no differences between Ctrl and Trpv4^–/–^ mice which corresponds with our previous study, where we also did not see any differences between the volume responses of Trpv4^–/–^ and Ctrl astrocytes *in situ* (Pivonkova et al., 2018). However, when we divided astrocytes into the aforementioned HRA and LRA subpopulations, we observed increased HRA swelling in Trpv4^–/–^ mice during hypoosmotic stress and, conversely, reduced HRA volume during OGD compared to controls. It is therefore apparent that experiments performed in acute brain slices or *in vivo* do not lead to as clear-cut results as *in vitro* experiments. The modest increase in HRA volume under conditions of hypoosmotic stress in Trpv4^–/–^ suggests that TRPV4 channels are involved to some extent in volume regulation. A similar increase was also observed in the work of Toft-Bertelsen et al. (2018) after the pharmacological block of TRPV4 channels. However, these authors also reported that TRPV4 inhibition did not affect RVD following astrocytic swelling induced by physiological stimulus. This implies that RVD persists independently of TRPV4 deletion and likely occurs via other mechanisms (for review see Wilson and Mongin, 2018). In contrast, Chmelova et al. (2019), using similar methodological approach that determines extracellular space volume as a parameter reflecting astrocyte swelling, observed less ECS shrinkage (less cell swelling) in Trpv4^–/–^ mice. Their measurements, however, were performed under OGD conditions, which represents a more severe insult. These results suggest that TRPV4 channels play a role in the overall swelling of neuropil cells.

Neuronal activity and local increase of [K^+^]_O_ causes astrocyte membrane depolarization during OGD, which activates astrocytic sodium-bicarbonate cotransporters (NBCs) (Köhler et al., 2018). Their activation then increases intracellular Na^+^ and simultaneously reduces the level of intracellular ATP in astrocytes (Everaerts et al., 2023), and therefore contributes to their swelling (Florence et al., 2012; Larsen and MacAulay, 2017). Interestingly, based on previous research Walch and Fiacco suggested in their review that the NBCs-dependent swelling occurs after approximately 10 min of the exposure to pathological conditions, and the first 10 min are NBCs-independent (Walch and Fiacco, 2022). This is consistent with the volume changes we detected in HRA from Trpv4^–/–^ mice, where during the first 10 min of the measurements the volume increase caused by OGD was only mild, however during another 10 min reached values similar to control group. Although astrocytes are known to be the main cells contributing to changes in ECS volume parameters (Walch and Fiacco, 2022), the contribution of other cell types must also be considered. It was previously demonstrated that neuronal TRPV4 channels modulate their excitability and even contribute to glutamate excitotoxicity and strong K^+^ release from neurons during ischemia (Shibasaki et al., 2007, 2015; Li et al., 2013; Jie et al., 2016). In the TRPV4 knockouts the excitability of neurons is decreased, and they require bigger stimulus to initiate similar response as wild type neurons, as was demonstrated in hippocampus (Shibasaki et al., 2007; Sucha et al., 2022). This can additionally contribute to the delay in the astrocyte swelling we detected in TRPV4 knockouts during OGD. Altogether, it would suggest that the TRPV4 channels play an important role in the swelling of astrocytes and their deletions causes reduction in said swelling. However, this effect is in time prevailed over by other mechanisms causing the cells to increase their volume. Similarly, Hoshi et al. (2018) observed reduced OGD-induced brain tissue swelling in TRPV4 knockouts or after treatment with the inhibitor HC-067047 suggesting its role in swelling rather than volume control.

TRPV4 expression is likely restricted to a specific population of astrocytes, and the number of expressing cells varies between brain regions (for review see Tureckova et al., 2023). Here we report that using scRT-qPCR we detected TRPV4 in only a small percentage of astrocytes (6%), but after exposure to pathological conditions we observed a significant difference in astrocyte volume changes in up to ∼30% (OGD) and ∼50% (H-100) of cells between controls and TRPV4 knockouts. The explanation may again be the fact that the volume changes observed in individual astrocytes result from the interaction of mechanisms occurring in the surrounding cell types. Moreover, the low positivity of astrocytes for TRPV4 expression detected in the RT-qPCR experiment may have both biological and technical reasons. Biologically, it is well-described that mRNA levels do not always accurately reflect protein levels (Nolan et al., 2006; Liu et al., 2016). This phenomenon is likely more pronounced in CNS cells, as many genes are encoded by long transcripts that need to be transported along lengthy processes to the endfeet and synapses, where they are translated into proteins (Sakers et al., 2017; Hafner et al., 2019). This causes a decoupling of transcription and translation. Additionally, differences in mRNA and protein detection rates may result from variations in decay halftime. Moreover, transcription occurs in transcription bursts, and the dynamics of these bursts may be gene-specific, with long intervals between individual bursts affecting detection rates (Lenstra et al., 2016; Brouwer and Lenstra, 2019; Wan et al., 2021). Technically, the low detection rate of TRPV4 transcripts might be explained by the limited sensitivity of reverse transcriptase and qPCR assay, as well as inevitable dilution steps in the RT-qPCR workflow. Another significant factor is the step of sample preparation. As the tissue needs to be dissociated into single-cell suspension, many astrocytic processes, where TRPV4 transcripts are likely localized (Tureckova et al., 2023), are lost, leading to decreased detection rates.

In addition, it is also important to consider the effect of global TRPV4 channel deletion on the expression of other channels and transporters that may also influence the observed volume changes. In this study, we selected several candidates, and those with altered expression were shown to include ClC2 ion channels and KCC1 transporters. Astrocytic swelling triggers outwardly rectifying Cl^–^ currents that help restore normal cellular volume (Parkerson and Sontheimer, 2004; Ernest et al., 2005). Numerous chloride channels have been described to participate in this process, including the VRAC, KCC and ClC channel families. We found a decreased level of expression of ClC2 channels in astrocytes from Trpv4^–/–^ mice. The ClC2 channels should be inhibited by the cytoskeleton during physiological conditions but are activated by the changes in cell shape and therefore by swelling (Kimelberg et al., 2006). The blockage of these channels leads to a decreased volume recovery after hypoosmotic challenge (Ernest et al., 2005; Formaggio et al., 2019). However, we found increased swelling of Trpv4^–/–^ HRA during hypoosmotic stress together with increased volume recovery in these cells, which speaks in favor of the limited involvement of ClC2 in cell volume restoration. Interestingly, ClC2 channels were rejected as participants in the active RVD, when their blocking failed to affect the RVD in human colonic cells. Similar results were shown also in ClC2 knockouts that showed RVD (in parotid acinar cells) similar to wild-type animals (Okada et al., 2019). Moreover, the role of ClC2 channels in astrocyte volume regulation of LRA and HRA was described by Benesova et al. (2012), who suggested that lower levels of ClC2 correspond with increased swelling of HRA. This might indicate the importance of ClC2 channels during astrocyte swelling, when they contribute to volume regulation. On the contrary, their role during volume recovery seems negligible.

Transporters such as NKCC, KCC or Na^+^/K^+^ ATPase, are associated with the removal of excessive K^+^ from the ECS, which is one of the essential functions of astrocytes (Murakami and Kurachi, 2016). This removal causes water influx into the astrocytes and thus cellular swelling, which is proportional to the astrocytic ability to buffer K^+^ (Neprasova et al., 2007; Risher et al., 2009; Kolenicova et al., 2020). Under ischemic conditions [K^+^]_O_ can reach 50 - 80 mM (Leis et al., 2005), which causes depolarization of the brain tissue (Illarionova et al., 2010; Du et al., 2018), and an immediate increase in the intracellular concentration of Ca^2+^ (Yaguchi and Nishizaki, 2010; Song and Gunnarson, 2012). We found a decreased expression of KCC1 transporters in mice lacking TRPV4. KCC transporters are, under physiological conditions, outwardly rectifying. However, under high [K^+^]_O_ the direction of transport reverses (Macaulay and Zeuthen, 2012). Therefore, less K^+^ enters the astrocytes with decreased KCC1 expression, and subsequently, less water follows. Even though the comparison of all astrocytes together did not show any significant effect of the TRPV4 deletion, when the cells were divided into LRA and HRA we saw that the deletion of TRPV4 channels caused a reduction of hyperkalemia-induced swelling in LRA, which corresponds with the decreased levels of KCC1. In addition to KCC1 and ClC2, we also observed a decrease in the expression of glutamate receptors, namely NMDA and mGluR6, in Trpv4^–/–^ by scRT-qPCR. Although glutamate receptor activation is not the major mechanism that causes astrocyte swelling, several papers have been published in which this phenomenon is described (Chan and Chu, 1990; Chan et al., 1990; Hansson, 1994; Yuan and Wang, 1996; Bender et al., 1998). Therefore, we hypothesize that reduced expression of NMDA or mGluR may contribute to the reduced swelling we observed in HRA in OGD. The last receptor whose expression is slightly altered due to TRPV4 deletion is GABA_B_, a heterodimeric G-protein coupled receptor composed of two subunits, B1 and B2. The activation of astroglial GABA_B_ receptors causes Ca^2+^ release from the endoplasmic reticulum via IP3 receptors. This mechanism most likely plays a role in the modulation of synaptic transmission (Charles et al., 2003; Ishibashi et al., 2019; Liu et al., 2022). To our knowledge, the direct effect of GABA_B_ receptor function on cerebral edema has not been studied. However, given its modulatory function, it is possible that its reduced expression under ischemic conditions indirectly influences the extent of the damage. Considering all the above mentioned mechanisms including global TRPV4 deletion as well as other independent processes contributing to the astrocyte volume changes we conclude that the interpretation of the role of TRPV4 channels in astrocyte swelling and volume recovery is at this point difficult and would require additional research.

### 4.4 The simultaneous deletion of AQP4 and TRPV4 channels limits astrocyte volume recovery after oxygen-glucose deprivation

Surprisingly, the simultaneous deletion of both AQP4 and TRPV4 channels did not alter astrocyte swelling. Our previously published data (Sucha et al., 2022) showed a protective effect of simultaneous deletion of both channels on the size of the lesion after permanent MCAO. However, it showed only small changes in the volume of extracellular space during OGD, indicating limited or no swelling of brain cells. Based on that data, we suggested a model of cell swelling which claims that the deletion of both channels leads to a slow swelling of the brain tissue during cerebral ischemia (Sucha et al., 2022). However, here we did not see any effect of double knockout on the swelling of astrocytes during hyperkalemia or OGD and only a minor volume increase of LRA during hypoosmotic stress. It is of interest that the protective effect of AQP4 deletion, which we observed in Aqp4^–/–^ mice during OGD seems to be reversed by the deletion of both channels. This would once again indicate the presence of other mechanisms that can substitute the role of said channels during astrocyte swelling and therefore their presence might not be required. Additionally, our previous model (Sucha et al., 2022) indicated was that the simultaneous deletion of both AQP4 and TRPV4 channels causes limited RVD. No differences in astrocyte volume recovery between Aqp4^–/–^/Trpv4^–/–^ and control mice were observed during exposure to hypoosmotic stress or hyperkalemia in this study, again suggesting that changes in astrocyte volume during both conditions may be independent of either channel and are controlled through different channels and transporters as suggested above. However, the deletion of AQP4 together with TRPV4 in astrocytes showed the inability of HRA to restore their volume after OGD which corresponds with our proposed model. It seems that even though both channels can be substituted during astrocyte swelling, they are crucial for volume recovery after OGD.

## 5 Conclusion

In our study, we investigated the effect of the deletion of AQP4 and TRPV4 channels on astrocytic volume changes induced by three models of ischemia-mimicking insults. We proved that cortical astrocytes do not respond to pathological stimuli with uniform changes in volume, and we identified two subpopulations responding with low or high-volume changes. From these subpopulations, the HRA were more affected by the loss of AQP4, TRPV4 or both. We confirmed that AQP4 is not essential for astrocyte swelling, but that other mechanisms that can compensate for the absence of AQP4 appear to be affected in OGD. Therefore, the deletion of AQP4 channels was protective and resulted in decreased swelling of astrocytes, during OGD. In addition, we showed that a lack of the TRPV4 channels does not lead to worsening of astrocyte swelling, as expected for its function in RVD, but instead resulted in a delay in astrocyte swelling. Surprisingly, the simultaneous deletion of both channels did not have a large effect on astrocyte volume in any of the three models used, only affecting the ability of astrocytes to return to their original volume after OGD. In addition, we have shown that knockout of AQP4 or TRPV4 can cause expression changes of other proteins, specifically glutamate receptors and ion channels, and thus the effect on the development of cerebral edema is not solely due to the specific deletion of these two channels.

## Data availability statement

The datasets presented in this study can be found in online repositories. The names of the repository/repositories and accession number(s) can be found in the article/Supplementary material.

## Ethics statement

The animal study was approved by the Institute of Experimental Medicine, Czech Academy of Sciences (Animal Care Committee on April 30, 2019; approval number 49/2019). The study was conducted in accordance with the local legislation and institutional requirements.

## Author contributions

ZH: Formal analysis, Investigation, Visualization, Writing – original draft, Data curation. LV: Data curation, Formal analysis, Investigation, Writing – review and editing. JK: Formal analysis, Investigation, Writing – original draft. MM: Writing – review and editing, Investigation. JT: Data curation, Supervision, Visualization, Writing – original draft. MK: Conceptualization, Supervision, Writing – review and editing, Funding acquisition. MA: Conceptualization, Project administration, Supervision, Writing – review and editing, Funding acquisition.

## Footnotes

1
http://www.cdb.riken.jp/arg/mutant%20mice%20list.html

2
https://fiji.sc/
