# Supplementary material for: The deletion of AQP4 and TRPV4 affects astrocyte swelling/volume recovery in response to ischemia-mimicking pathologies
Source: Front Cell Neurosci. 2024 May 15;18:1393751. doi: 10.3389/fncel.2024.1393751 (PMC11138210; doi:10.3389/fncel.2024.1393751)
Supplement: Supplementary file 2 [file Table_2.PDF]

Supplementary table 2: Average values and statistics (knock-out strains compared to the apropriate Ctrl).

Strain	Time (min)	H-100 Mean ± SEM	H-100 N	H-100 P value (DF, F value)	$50mM K^+$ Mean ± SEM	$50mM K^+$ N	$50mM K^+$ P value (DF, F value)	OGD Mean ± SEM	OGD N	OGD P value (DF, F value)
Ctrl	0	100.00 ± 00.00	18		100.00 ± 00.00	15		100.00 ± 00.00	16	
	10	189.76 ± 13.36			326.15 ± 23.66			145.54 ± 9.12		
	20	218.93 ± 20.19			380.36 ± 37.73			149.66 ± 13.02		
	30	160.47 ± 12.35			262.18 ± 23.77			138.47 ± 8.79		
	40	149. 25 ± 11.68			227.42 ± 20.48			133.94 ± 9.51		
	recovery	-26.67 ± 2.85			-37.67 ± 3.14			-2.78 ± 7.38		
$Aqp4^{-/-}$	0	100.00 ± 00.00	21	p > 0.9999 (185, 27.61)	100.00 ± 00.00	16	p > 0.9999 (145, 25.41)	100.00 ± 00.00	16	p > 0.9999 (150, 6.325)
	10	174.09 ± 8.86		p > 0.9999 (185, 27.61)	258.92 ± 29.55		p = 0.3208 (145, 25.41)	112.97 ± 3.30		p = 0.0141 (150, 6.325)
	20	195.41 ± 11.20		p > 0.9999 (185, 27.61)	296.02 ± 39.98		p = 0.1032 (145, 25.41)	110.58 ± 3.38		p = 0.0018 (150, 6.325)
	30	150.60 ± 9.75		p > 0.9999 (185, 27.61)	208.27 ± 23.78		p = 0.6840 (145, 25.41)	122.26 ± 5.91		p = 0.6650 (150, 6.325)
	40	146.16 ± 9.62		p > 0.9999 (185, 27.61)	185.33 ± 21.02		p > 0.9999 (145, 25.41)	127.63 ± 9.87		p > 0.9999 (150, 6.325)
	recovery	-25.08 ± 2.79		p = 0.9670 (79, 2.915)	-33.65 ± 3.22		p = 0.6476 (57, 1.715)	19.00 ± 6.61		p = 0.0827 (69, 3.460)
$Trpv4^{-/-}$	0	100.00 ± 00.00	27	p > 0.9999 (215, 31.28)	100.00 ± 00.00	14	p > 0.9999 (135, 15.72)	100.00 ± 00.00	19	p > 0.9999 (165, 7.241)
	10	193.76 ± 14.28		p > 0.9999 (215, 31.28)	277.46 ± 30.78		p > 0.9999 (135, 15.72)	122.94 ± 4.92		p = 0.2752 (165, 7.241)
	20	213.31 ± 15.81		p > 0.9999 (215, 31.28)	360. 00 ± 77.90		p > 0.9999 (135, 15.72)	135.75 ± 9.42		p = 0.3403 (165, 7.241)
	30	139.68 ± 6.87		p = 0.9902 (215, 31.28)	259.92 ± 45.68		p > 0.9999 (135, 15.72)	127.71 ± 9.24		p > 0.9999 (165, 7.241)
	40	134.68 ± 8.00		p > 0.9999 (215, 31.28)	222.39 ± 35.73		p > 0.9999 (135, 15.72)	131.58 ± 9.95		p > 0.9999 (165, 7.241)
	recovery	-35.16 ± 3.12		p = 0.1094 (79, 2.915)	-27.97 ± 2.03		p = 0.0709 (57, 1.715)	3.89 ± 5.63		p = 0.8209 (69, 3.460)
$Aqp4^{-/-}/Trpv4^{-/-}$	0	100.00 ± 00.00	17	p > 0.9999 (165, 25.61)	100.00 ± 00.00	16	p > 0.9999 (145, 38.90)	100.00 ± 00.00	22	p > 0.9999 (180, 5.348)
	10	173.41 ± 9.51		p > 0.9999 (165, 25.61)	298.91 ± 23.24		p > 0.9999 (145, 38.90)	143.39 ± 13.50		p > 0.9999 (180, 5.348)
	20	207.08 ± 17.67		p > 0.9999 (165, 25.61)	320.89 ± 29.98		p = 0.2886 (145, 38.90)	153.51 ± 16.92		p > 0.9999 (180, 5.348)
	30	137.44 ± 9.22		p = 0.7713 (165, 25.61)	235.65 ± 16.86		p > 0.9999 (145, 38.90)	178.67 ± 20.75		p = 0.3230 (180, 5.348)
	40	133.68 ± 9.15		p > 0.9999 (165, 25.61)	204.87 ± 14.33		p > 0.9999 (145, 38.90)	188.54 ± 23.86		p = 0.0621 (180, 5.348)
	recovery	-33.62 ± 2.79		p = 0.3025 (79, 2.915)	-33.13 ± 3.09		p = 0.5618 (57, 1.715)	20.30 ± 6.73		p = 0.0177 (69, 3.460)

Abbreviations: $Aqp4^{-/-}$, Aquaporin 4-deficient; $Aqp4^{-/-}/Trpv4^{-/-}$, Aquaporin 4-, Transient Receptor Potential Vanilloid 4-deficient; Ctrl, control; DF, degrees of freedom; H-100, hypoosmotic stress; OGD, oxygen-glucose deprivation; $Trpv4^{-/-}$, Transient Receptor Potential Vanilloid 4-deficient; $50mM K^+$, hyperkalemia.
