# Supplementary material for: The deletion of AQP4 and TRPV4 affects astrocyte swelling/volume recovery in response to ischemia-mimicking pathologies
Source: Front Cell Neurosci. 2024 May 15;18:1393751. doi: 10.3389/fncel.2024.1393751 (PMC11138210; doi:10.3389/fncel.2024.1393751)
Supplement: Supplementary file 3 [file Table_3.PDF]

Supplementary table 3: Average values and statistics of LRA (knock-out strains compared to the apropriate Ctrl).

Strain	Time (min)	H-100 Mean ± SEM	H-100 N	H-100 P value (DF, F value)	50mM K^+ Mean ± SEM	50mM K^+ N	50mM K^+ P value (DF, F value)	OGD Mean ± SEM	OGD N	OGD P value (DF, F value)
Ctrl	0	100.00 ± 00.00	10		100.00 ± 00.00	6		100.00 ± 00.00	11	
	10	142.00 ± 7.63			227.33 ± 15.30			122.06 ± 4.43		
	20	148.22 ± 10.46			233.00 ± 13.32			115.00 ± 7.64		
	30	117.24 ± 6.49			179.82 ± 12.58			119.22 ± 6.36		
	40	108.20 ± 6.85			170.34 ± 6.06			118.94 ± 7.92		
	recovery	-25.44 ± 5.06			-25.98 ± 4.09			6.23 ± 8.29		
$Aqp4^{-/-}$	0	100.00 ± 00.00	14	p > 0.9999 (110, 17.89)	100.00 ± 00.00	10	p > 0.9999 (70, 30.06)	100.00 ± 00.00	16	p > 0.9999 (125, 6.380)
	10	150.48 ± 7.55		p > 0.9999 (110, 17.89)	181.61 ± 12.63		p = 0.0331 (70, 30.06)	111.81 ± 3.29		p = 0.6168 (125, 6.380)
	20	168.20 ± 8.46		p = 0.3524 (110, 17.89)	185.64 ± 14.96		p = 0.0249 (70, 30.06)	109.01 ± 3.18		p > 0.9999 (125, 6.380)
	30	130.59 ± 9.10		p > 0.9999 (110, 17.89)	146.13 ± 10.51		p = 0.2144 (70, 30.06)	117.59 ± 3.85		p > 0.9999 (125, 6.380)
	40	127.53 ± 9.14		p = 0.3999 (110, 17.89)	126.98 ± 9.06		p = 0.0491 (70, 30.06)	123.32 ± 5.41		p > 0.9999 (125, 6.380)
	recovery	-24.41 ± 3.88		p = 0.9939 (52, 1.516)	-29.56 ± 4.58		p = 0.8415 (26, 0.2376)	11.27 ± 4.76		p = 0.8930 (48, 0.3698)
$Trpv4^{-/-}$	0	100.00 ± 00.00	19	p > 0.9999 (135, 29.52)	100.00 ± 00.00	7	p > 0.9999 (55, 18.11)	100.00 ± 00.00	16	p > 0.9999 (125, 4.464)
	10	162.60 ± 5.35		p = 0.1413 (135, 29.52)	170.17 ± 17.69		p = 0.0255 (55, 18.11)	125.54 ± 5.59		p > 0.9999 (125, 4.464)
	20	168.19 ± 7.11		p = 0.1662 (135, 29.52)	168.82 ± 21.28		p = 0.0091 (55, 18.11)	116.11 ± 6.02		p > 0.9999 (125, 4.464)
	30	125.38 ± 5.69		p > 0.9999 (135, 29.52)	142.41 ± 16.32		p = 0.3072 (55, 18.11)	114.35 ± 6.12		p > 0.9999 (125, 4.464)
	40	118.19 ± 6.63		p > 0.9999 (135, 29.52)	122.43 ± 12.61		p = 0.0886 (55, 18.11)	119.44 ± 7.61		p > 0.9999 (125, 4.464)
	recovery	-29.37 ± 2.78		p = 0.7475 (52, 1.516)	-25.98 ± 2.85		p > 0.9999 (26, 0.2376)	4.00 ± 5.98		p = 0.9883 (48, 0.3698)
$Aqp4^{-/-}/Trpv4^{-/-}$	0	100.00 ± 00.00	13	p > 0.9999 (105, 27.33)	100.00 ± 00.00	7	p > 0.9999 (55, 57.64)	100.00 ± 00.00	12	p > 0.9999 (105, 3.114)
	10	161.12 ± 8.73		p = 3404 (105, 27.33)	216.44 ± 9.69		p > 0.9999 (55, 57.64)	110.93 ± 4.74		p = 0.8159 (105, 3.114)
	20	179.09 ± 10.92		p = 0.0181 (105, 27.33)	216.77 ± 9.79		p > 0.9999 (55, 57.64)	104.11 ± 5.12		p = 0.8608 (105, 3.114)
	30	119.25 ± 4.93		p > 0.9999 (105, 27.33)	187.28 ± 8.24		p > 0.9999 (55, 57.64)	112.27 ± 6.09		p > 0.9999 (105, 3.114)
	40	116.81 ± 6.42		p > 0.9999 (105, 27.33)	159.74 ± 8.52		p > 0.9999 (55, 57.64)	112.20 ± 7.06		p > 0.9999 (105, 3.114)
	recovery	-34.05 ± 2.28		p = 0.2499 (52, 1.516)	-26.28 ± 2.30		p > 0.9999 (26, 0.2376)	11.48 ± 6.56		p = 0.9068 (48, 0.3698)

Abbreviations: $Aqp4^{-/-}$, Aquaporin 4-deficient; $Aqp4^{-/-}/Trpv4^{-/-}$, Aquaporin 4-, Transient Receptor Potential Vanilloid 4-deficient; Ctrl, control; DF, degrees of freedom; H-100, hypoosmotic stress; LRA, low-responding astrocytes; OGD, oxygen-glucose deprivation; $Trpv4^{-/-}$, Transient Receptor Potential Vanilloid 4-deficient; 50mM K^+, hyperkalemia.
