# Supplementary material for: The deletion of AQP4 and TRPV4 affects astrocyte swelling/volume recovery in response to ischemia-mimicking pathologies
Source: Front Cell Neurosci. 2024 May 15;18:1393751. doi: 10.3389/fncel.2024.1393751 (PMC11138210; doi:10.3389/fncel.2024.1393751)
Supplement: Supplementary file 4 [file Table_4.PDF]

Supplementary table 4: Average values and statistics of HRA (knock-out strains compared to the apropriate Ctrl).

Strain	Time (min)	H-100			50mM K^+			OGD		
		Mean ± SEM	N	P value (DF, F value)	Mean ± SEM	N	P value (DF, F value)	Mean ± SEM	N	P value (DF, F value)
Ctrl	0	100.00 ± 00.00	8		100.00 ± 00.00	8		100.00 ± 00.00	5	
	10	249.45 ± 13.98			328.25 ± 18.46			191.00 ± 15.02		
	20	279.40 ± 10.78			360.01 ± 11.57			215.66 ± 20.84		
	30	214.50 ± 14.51			235.67 ± 7.06			177.15 ± 11.18		
	40	200.58 ± 12.96			196.57 ± 11.04			177.42 ± 12.31		
	recovery	-28.22 ± 3.64			-45.00 ± 3.43			-13.38 ± 13.59		
$Aqp4^{-/-}$	0	100.00 ± 00.00	7	$p > 0.9999$ (65, 47.52)	100.00 ± 00.00	6	$p > 0.9999$ (65, 93.20)			
	10	221.31 ± 7.50		$p = 0.6204$ (65, 47.52)	387.79 ± 34.54		$p = 0.1087$ (65, 93.20)			
	20	249.84 ± 17.95		$p = 0.5320$ (65, 47.52)	479.99 ± 35.86		$p < 0.0001$ (65, 93.20)			
	30	190.63 ± 16.77		$p = 0.9539$ (65, 47.52)	311.82 ± 27.57		$p = 0.0187$ (65, 93.20)			
	40	183.42 ± 17.44		$p > 0.9999$ (65, 47.52)	282.58 ± 16.22		$p = 0.0058$ (65, 93.20)			
	recovery	-26.43 ± 4.44		$p = 0.9931$ (23, 3.306)	-40.47 ± 2.28		$p = 0.7525$ (27, 1.226)			
$Trpv4^{-/-}$	0	100.00 ± 00.00	8	$p > 0.9999$ (70, 29.01)	100.00 ± 00.00	7	$p > 0.9999$ (70, 71.17)	100.00 ± 00.00	3	$p > 0.9999$ (30, 14.93)
	10	263.43 ± 41.59		$p > 0.9999$ (70, 29.01)	358.82 ± 24.87		$p > 0.9999$ (70, 71.17)	109.05 ± 3.25		$p = 0.0034$ (30, 14.93)
	20	366.08 ± 42.32		$p = 0.0326$ (70, 29.01)	414.29 ± 34.47		$p = 0.2151$ (70, 71.17)	192.51 ± 7.36		$p > 0.9999$ (30, 14.93)
	30	173.13 ± 16.01		$p = 0.9260$ (70, 29.01)	301.51 ± 29.18		$p = 0.0739$ (70, 71.17)	198.95 ± 18.85		$p > 0.9999$ (30, 14.93)
	40	173.86 ± 17.67		$p > 0.9999$ (70, 29.01)	268.52 ± 28.72		$p = 0.0398$ (70, 71.17)	196.30 ± 29.02		$p > 0.9999$ (30, 14.93)
	recovery	-48.91 ± 7.10		$p = 0.0401$ (23, 3.306)	-35.74 ± 3.41		$p = 0.2092$ (27, 1.226)	3.34 ± 19.37		$p > 0.7406$ (15, 3.478)
$Aqp4^{-/-}/Trpv4^{-/-}$	0	100.00 ± 00.00	4	$p > 0.9999$ (50, 37.10)	100.00 ± 00.00	9	$p > 0.9999$ (80, 82.50)	100.00 ± 00.00	10	$p > 0.9999$ (65, 7.504)
	10	213.36 ± 19.05		$p = 0.5777$ (50, 37.10)	315.90 ± 18.11		$p > 0.9999$ (80, 82.50)	182.33 ± 24.37		$p > 0.9999$ (65, 7.504)
	20	298.04 ± 43.84		$p > 0.9999$ (50, 37.10)	368.42 ± 28.21		$p > 0.9999$ (80, 82.50)	212.79 ± 26.74		$p > 0.9999$ (65, 7.504)
	30	196.53 ± 9.30		$p > 0.9999$ (50, 37.10)	287.62 ± 20.10		$p = 0.1188$ (80, 82.50)	258.35 ± 29.37		$p = 0.1875$ (65, 7.504)
	40	188.49 ± 7.15		$p > 0.9999$ (50, 37.10)	237.95 ± 19.70		$p = 0.3502$ (80, 82.50)	280.16 ± 33.76		$p = 0.0457$ (65, 7.504)
	recovery	-32.24 ± 10.40		$p = 0.9567$ (23, 3.306)	-24.65 ± 4.38		$p = 0.9877$ (27, 1.226)	35.12 ± 11.68		$p = 0.0412$ (15, 3.478)

Abbreviations: $Aqp4^{-/-}$, Aquaporin 4-deficient; $Aqp4^{-/-}/Trpv4^{-/-}$, Aquaporin 4-, Transient Receptor Potential Vanilloid 4-deficient; Ctrl, control; DF, degrees of freedom; H-100, hypoosmotic stress; HRA, high-responding astrocytes; OGD, oxygen-glucose deprivation; $Trpv4^{-/-}$, Transient Receptor Potential Vanilloid 4-deficient; 50mM K^+, hyperkalemia.
