# Supplementary material for: The deletion of AQP4 and TRPV4 affects astrocyte swelling/volume recovery in response to ischemia-mimicking pathologies
Source: Front Cell Neurosci. 2024 May 15;18:1393751. doi: 10.3389/fncel.2024.1393751 (PMC11138210; doi:10.3389/fncel.2024.1393751)
Supplement: Supplementary file 5 [file Data_Sheet_1.PDF]

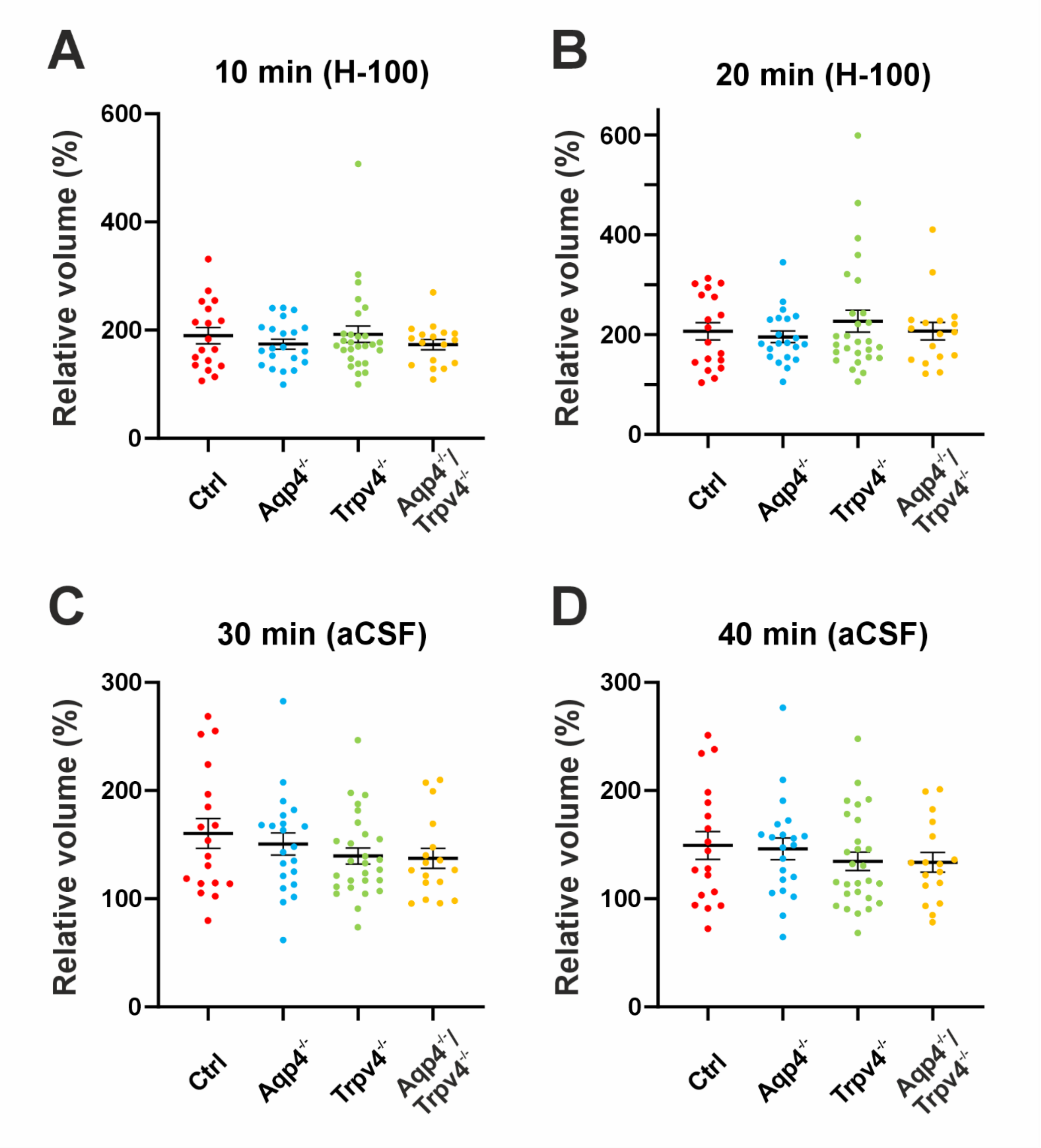


**Supplementary figure 1: Swelling of the soma of cortical astrocytes during hypoosmotis stress.** Individual data points and mean ± SEM showing swelling of astrocyte soma during 10 (**A**) and 20 (**B**) min of hypoosmotic stress. This was followed by 20 min washout in aCSF (**C**, **D**). Note that there were no differences between the experimental groups.

Abbreviations: aCSF, artificial cerebrospinal fluid; Aqp4$^{-/-}$, Aquaporin 4 knock-out; Aqp4$^{-/-}$/Trpv4$^{-/-}$, Aquaporin 4 and Transient Receptor Potential Vanilloid 4 double knock-out; Ctrl, control; H-100, hypoosmotic stress (200 mOsm hypotonic solution); Trpv4$^{-/-}$, Transient Receptor Potential Vanilloid 4 knock-out.
