# Supplementary material for: The deletion of AQP4 and TRPV4 affects astrocyte swelling/volume recovery in response to ischemia-mimicking pathologies
Source: Front Cell Neurosci. 2024 May 15;18:1393751. doi: 10.3389/fncel.2024.1393751 (PMC11138210; doi:10.3389/fncel.2024.1393751)
Supplement: Supplementary file 7 [file Data_Sheet_3.PDF]

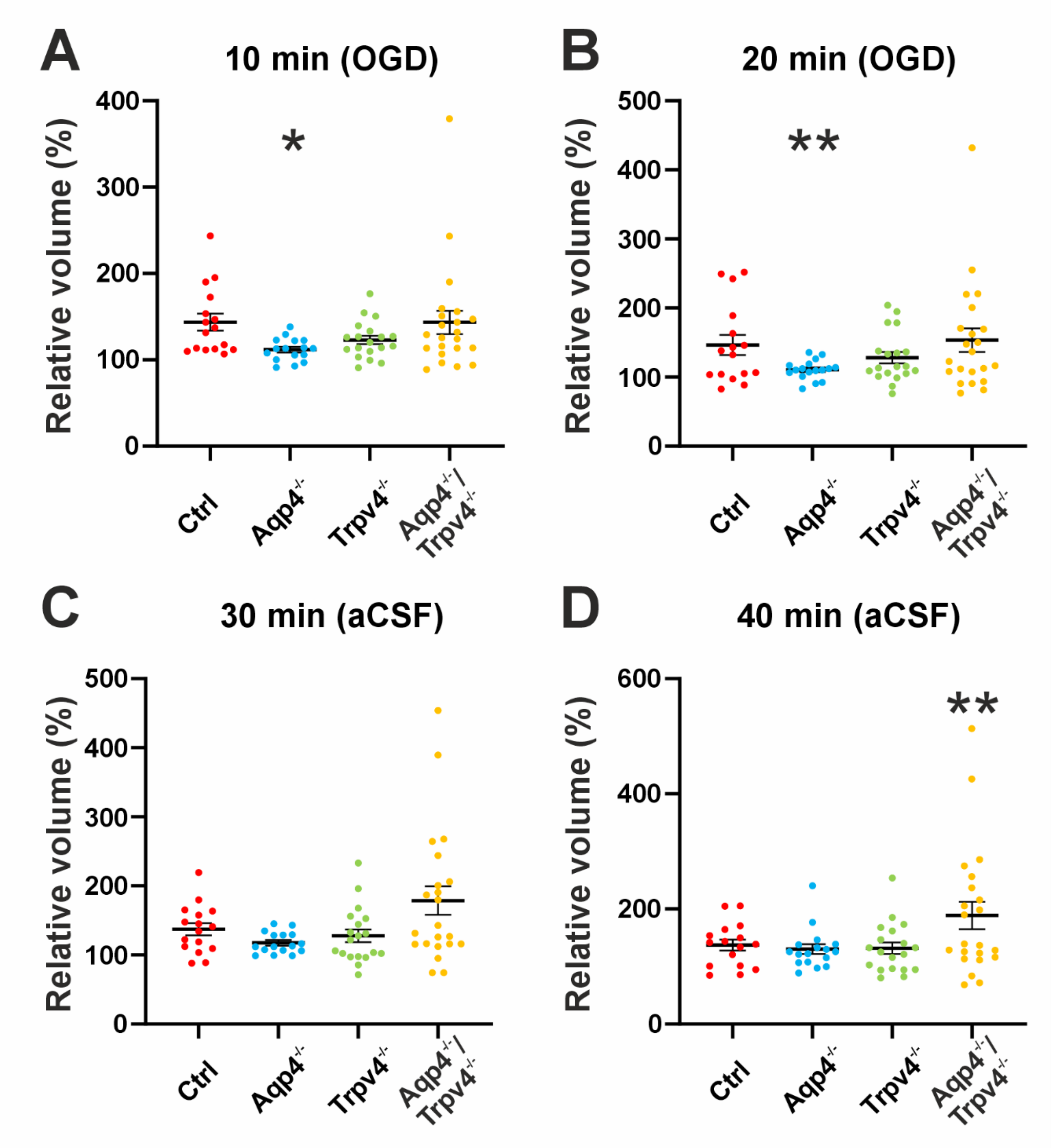

Supplementary figure 3: Swelling of the soma of cortical astrocytes during OGD. Individual data points and mean ± SEM showing swelling of astrocyte soma during 10 (**A**) and 20 (**B**) min of OGD. This was followed by 20 min washout in aCSF (**C**, **D**). Note that astrocytes from Aqp4$^{-/-}$ mice reached significantly smaller volume after10 and 20 min of OGD, compared to Ctrl (* $p < 0.05$; ** $p < 0.01$), and Aqp4$^{-/-}$/Trpv4$^{-/-}$ astrocytes were unable to restore their volume during washout (** $p < 0.01$).

Abbreviations: aCSF, artificial cerebrospinal fluid; Aqp4$^{-/-}$, Aquaporin 4 knock-out; Aqp4$^{-/-}$/Trpv4$^{-/-}$, Aquaporin 4 and Transient Receptor Potential Vanilloid 4 double knock-out; Ctrl, control; OGD, oxygen-glucose deprivation; Trpv4$^{-/-}$, Transient Receptor Potential Vanilloid 4 knock-out.
