# Supplementary material for: The deletion of AQP4 and TRPV4 affects astrocyte swelling/volume recovery in response to ischemia-mimicking pathologies
Source: Front Cell Neurosci. 2024 May 15;18:1393751. doi: 10.3389/fncel.2024.1393751 (PMC11138210; doi:10.3389/fncel.2024.1393751)
Supplement: Supplementary file 8 [file Data_Sheet_4.PDF]

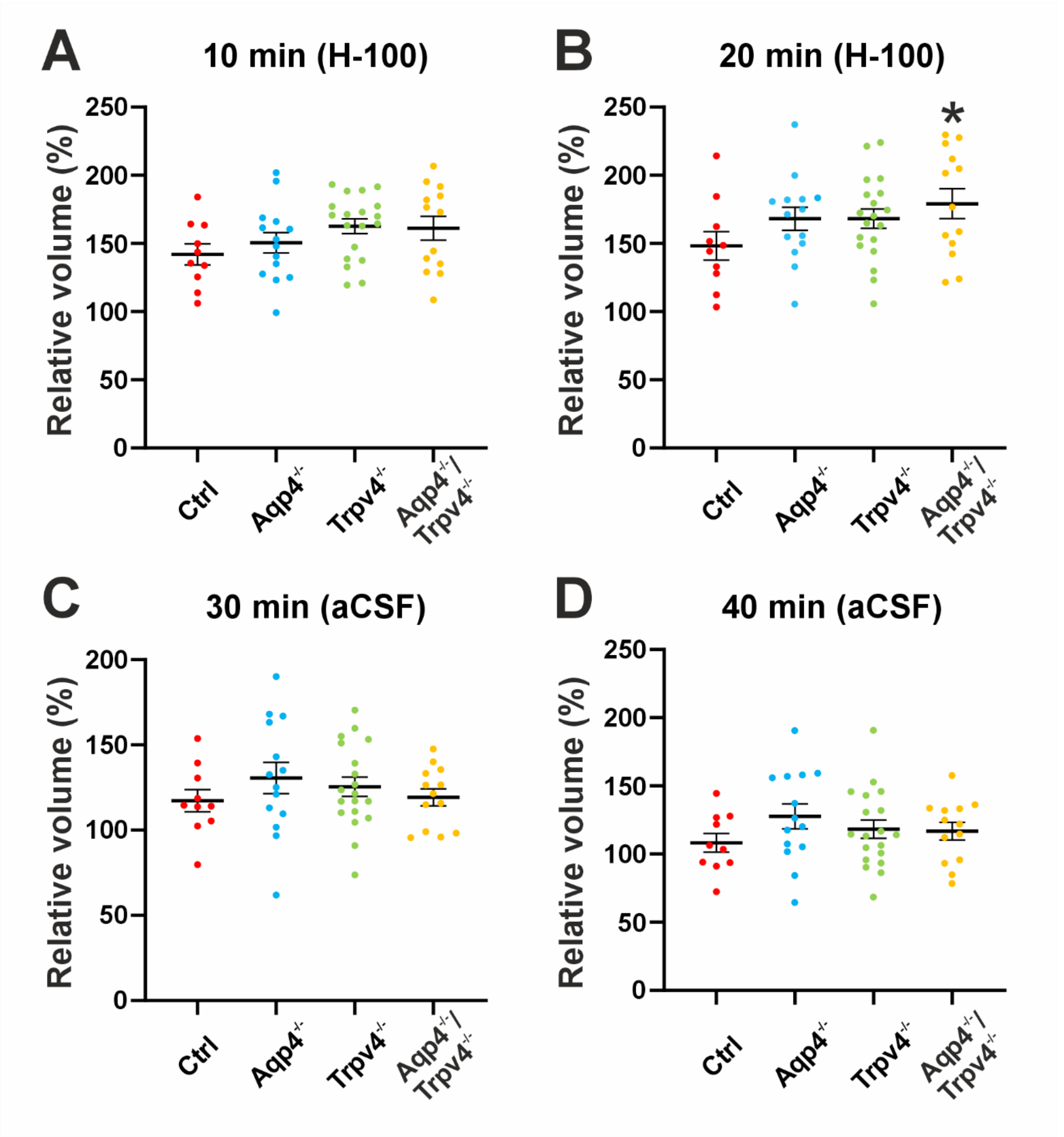

Supplementary figure 4: Swelling of the soma of cortical low-responding astrocytes during hypoosmotis stress. Individual data points and mean ± SEM showing swelling of LRA soma during 10 (**A**) and 20 (**B**) min of hypoosmotic stress. This was followed by 20 min washout in aCSF (**C**, **D**). Note that the LRA from $Aqp4^{-/-}/Trpv4^{-/-}$ reached significantly higher volume after 20 min exposure to H-100, compared to Ctrl (* $p < 0.05$).

Abbreviations: aCSF, artificial cerebrospinal fluid; $Aqp4^{-/-}$, Aquaporin 4 knock-out; $Aqp4^{-/-}/Trpv4^{-/-}$, Aquaporin 4 and Transient Receptor Potential Vanilloid 4 double knock-out; Ctrl, control; H-100, hypoosmotic stress (200 mOsm hypotonic solution); LRA, low-responding astrocytes; $Trpv4^{-/-}$, Transient Receptor Potential Vanilloid 4 knock-out.
