# Supplementary material for: The deletion of AQP4 and TRPV4 affects astrocyte swelling/volume recovery in response to ischemia-mimicking pathologies
Source: Front Cell Neurosci. 2024 May 15;18:1393751. doi: 10.3389/fncel.2024.1393751 (PMC11138210; doi:10.3389/fncel.2024.1393751)
Supplement: Supplementary file 9 [file Data_Sheet_5.PDF]

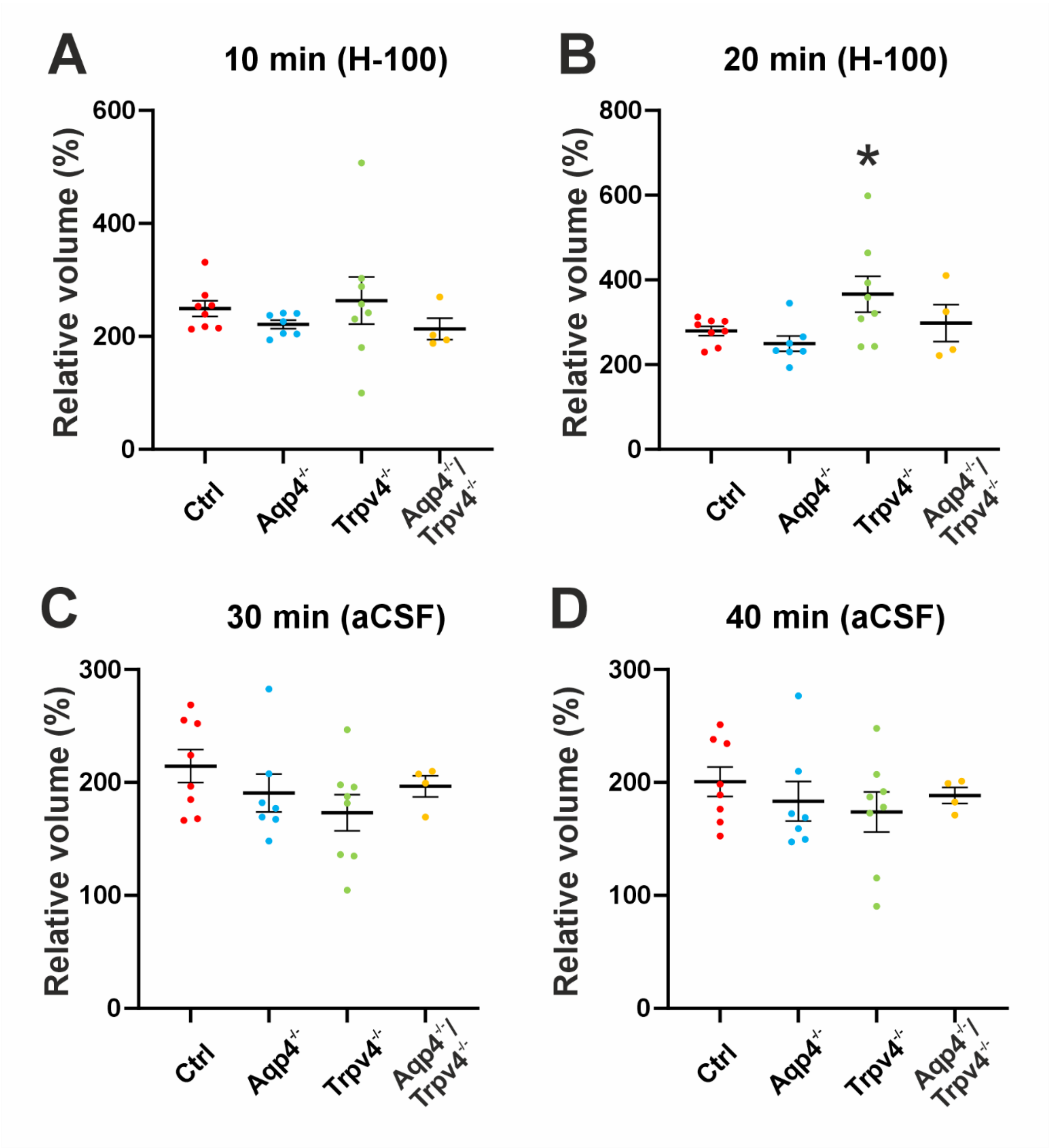

Supplementary figure 5: Swelling of the soma of cortical high-responding astrocytes during hypoosmotis stress. Individual data points and mean ± SEM showing swelling of HRA soma during 10 (**A**) and 20 (**B**) min of hypoosmotic stress. This was followed by 20 min washout in aCSF (**C**, **D**). Note that the HRA from Trpv4$^{-/-}$ reached significantly higher volume after 20 min exposure to H-100, compared to Ctrl (* $p < 0.05$).

Abbreviations: aCSF, artificial cerebrospinal fluid; Aqp4$^{-/-}$, Aquaporin 4 knock-out; Aqp4$^{-/-}$/Trpv4$^{-/-}$, Aquaporin 4 and Transient Receptor Potential Vanilloid 4 double knock-out; Ctrl, control; H-100, hypoosmotic stress (200 mOsm hypotonic solution); HRA, high-responding astrocytes; Trpv4$^{-/-}$, Transient Receptor Potential Vanilloid 4 knock-out.
