# Supplementary material for: The deletion of AQP4 and TRPV4 affects astrocyte swelling/volume recovery in response to ischemia-mimicking pathologies
Source: Front Cell Neurosci. 2024 May 15;18:1393751. doi: 10.3389/fncel.2024.1393751 (PMC11138210; doi:10.3389/fncel.2024.1393751)
Supplement: Supplementary file 10 [file Data_Sheet_6.PDF]

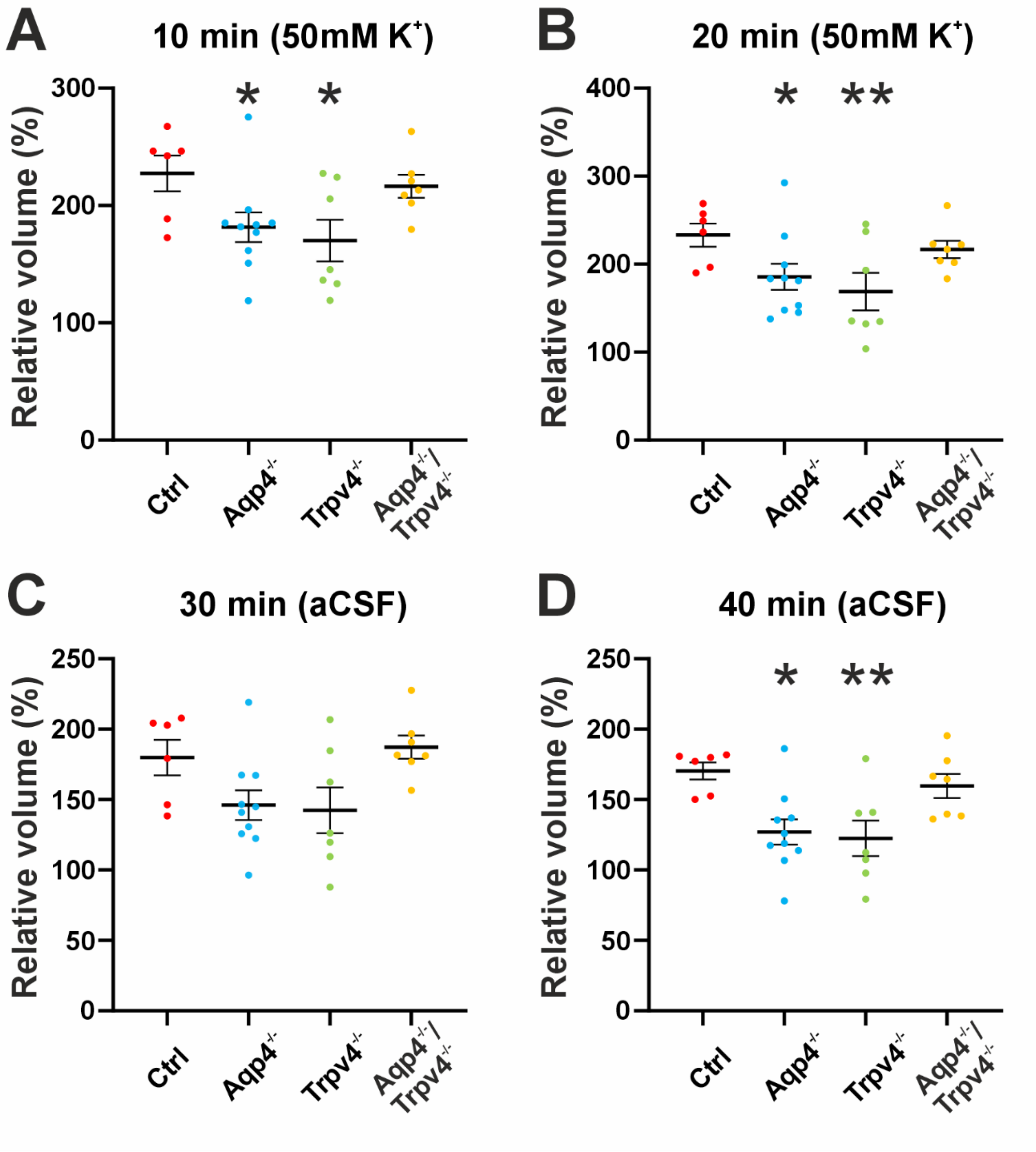

Supplementary figure 6: Swelling of the soma of cortical low-responding astrocytes during hyperkalemia. Individual data points and mean ± SEM showing swelling of LRA soma during 10 (**A**) and 20 (**B**) min of hyperkalemia. This was followed by 20 min washout in aCSF (**C**, **D**). Interestingly, both the LRA from Aqp4$^{-/-}$ and Trpv4$^{-/-}$ mice reached significantly smaller volume after 10 and 20 min exposure to 50mM K^+, and after 20 min of washout in aCSF, compared to Ctrl (* $p < 0.05$; ** $p < 0.01$).

Abbreviations: aCSF, artificial cerebrospinal fluid; Aqp4$^{-/-}$, Aquaporin 4 knock-out; Aqp4$^{-/-}$/Trpv4$^{-/-}$, Aquaporin 4 and Transient Receptor Potential Vanilloid 4 double knock-out; Ctrl, control; LRA, low-responding astrocytes; Trpv4$^{-/-}$, Transient Receptor Potential Vanilloid 4 knock-out; 50mM K^+, hyperkalemic solution (aCSF with elevated K^+ concentration).
