# Supplementary material for: The deletion of AQP4 and TRPV4 affects astrocyte swelling/volume recovery in response to ischemia-mimicking pathologies
Source: Front Cell Neurosci. 2024 May 15;18:1393751. doi: 10.3389/fncel.2024.1393751 (PMC11138210; doi:10.3389/fncel.2024.1393751)
Supplement: Supplementary file 11 [file Data_Sheet_7.PDF]

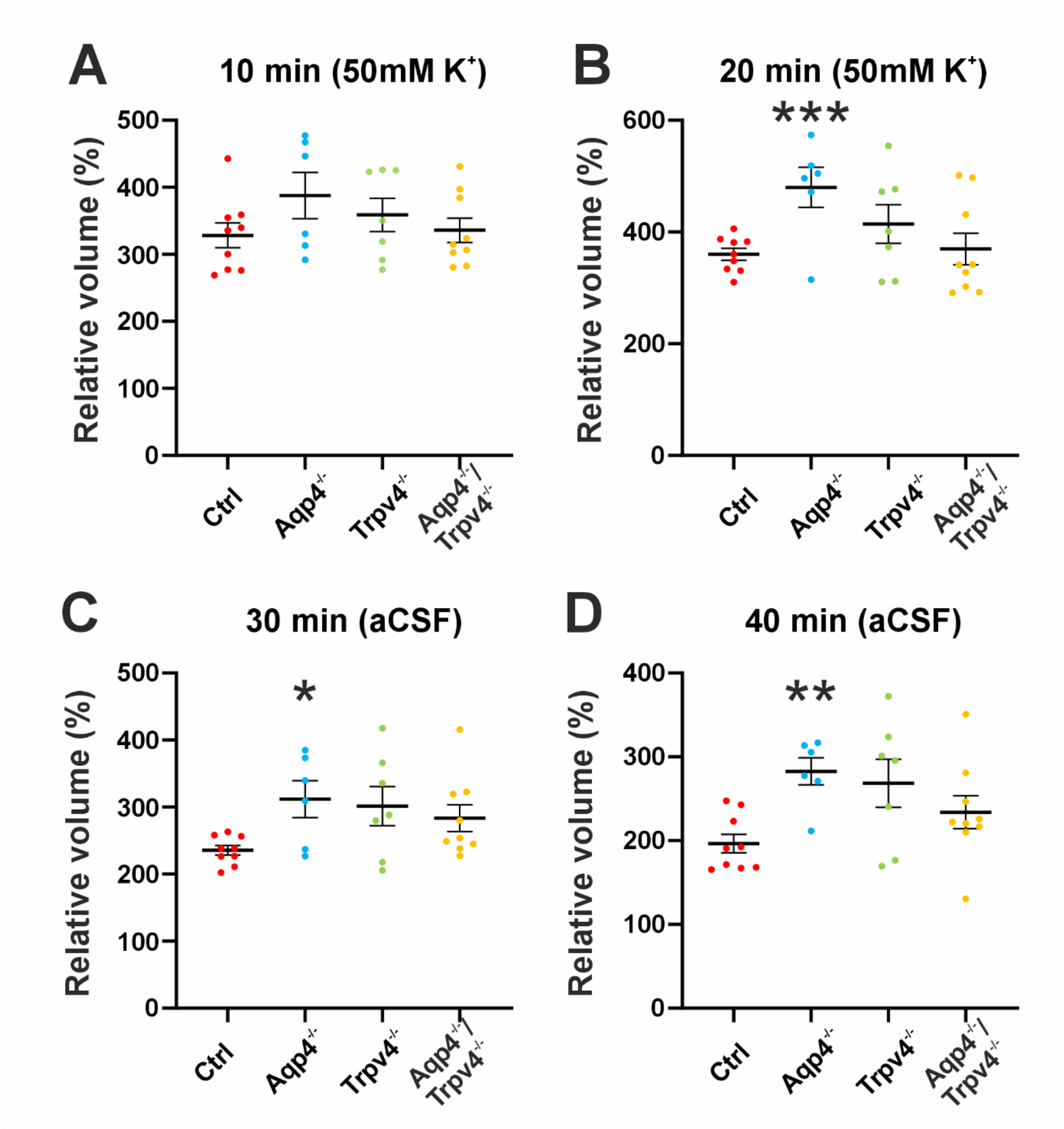

Supplementary figure 7: Swelling of the soma of cortical high-responding astrocytes during hyperkalemia. Individual data points and mean ± SEM showing swelling of HRA soma during 10 (**A**) and 20 (**B**) min of hyperkalemia, followed by 20 min washout in aCSF (**C**, **D**). Note that the HRA from $Aqp4^{-/-}$ mice reached significantly higher volume after 20 min exposure to 50mM K^+, and after 10 and 20 min of washout in aCSF, compared to Ctrl (* $p < 0.05$; ** $p < 0.01$; *** $p < 0.001$).

Abbreviations: aCSF, artificial cerebrospinal fluid; $Aqp4^{-/-}$, Aquaporin 4 knock-out; $Aqp4^{-/-}/Trpv4^{-/-}$, Aquaporin 4 and Transient Receptor Potential Vanilloid 4 double knock-out; Ctrl, control; HRA, high-responding astrocytes; $Trpv4^{-/-}$, Transient Receptor Potential Vanilloid 4 knock-out; 50mM K^+, hyperkalemic solution (aCSF with elevated K^+ concentration).
