# Supplementary material for: The deletion of AQP4 and TRPV4 affects astrocyte swelling/volume recovery in response to ischemia-mimicking pathologies
Source: Front Cell Neurosci. 2024 May 15;18:1393751. doi: 10.3389/fncel.2024.1393751 (PMC11138210; doi:10.3389/fncel.2024.1393751)
Supplement: Supplementary file 12 [file Data_Sheet_8.PDF]

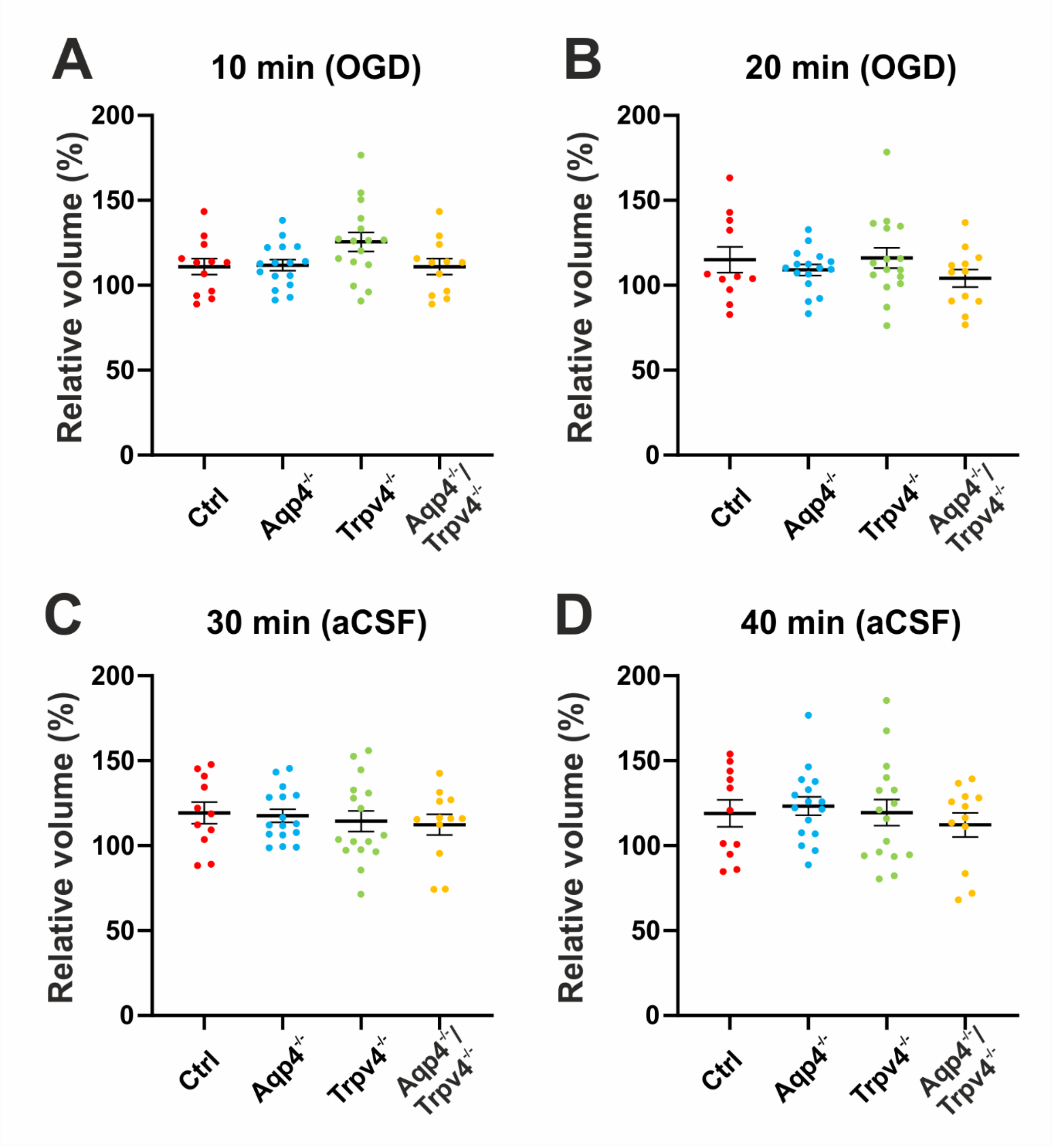

Supplementary figure 8: Swelling of the soma of cortical low-responding astrocytes during oxygen-glucose deprivation. Individual data points and mean ± SEM showing swelling of LRA soma during 10 (**A**) and 20 (**B**) min of OGD. This was followed by 20 min washout in aCSF (**C**, **D**).

Abbreviations: aCSF, artificial cerebrospinal fluid; $Aqp4^{-/-}$, Aquaporin 4 knock-out; $Aqp4^{-/-}/Trpv4^{-/-}$, Aquaporin 4 and Transient Receptor Potential Vanilloid 4 double knock-out; Ctrl, control; LRA, low-responding astrocytes; OGD, oxygen-glucose deprivation; $Trpv4^{-/-}$, Transient Receptor Potential Vanilloid 4 knock-out.
